# Adaptation to drought is coupled with slow growth, but independent from phenology in marginal silver fir (*Abies alba* Mill.) populations

**DOI:** 10.1111/eva.13029

**Published:** 2020-06-17

**Authors:** Katalin Csilléry, Nina Buchmann, Bruno Fady

**Affiliations:** ^1^ Department of Evolutionary Biology and Environmental Studies University of Zürich Zürich Switzerland; ^2^ Biodiversity & Conservation Biology Swiss Federal Research Institute WSL Birmensdorf Switzerland; ^3^ Institute of Agricultural Sciences ETH Zürich Zürich Switzerland; ^4^ INRA Ecology of Mediterranean Forests (URFM) UR629 Avignon France

**Keywords:** adaptive divergence, assisted migration, climate change, demography, drought tolerance, phenology, quantitative genetics, δ^13^C

## Abstract

Drought is one of the most important selection pressures for forest trees in the context of climate change. Yet, the different evolutionary mechanisms, and their environmental drivers, by which certain populations become more drought tolerant than others is still little understood. We studied adaptation to drought in 16 silver fir (*Abies alba* Mill.) populations from the French Mediterranean Alps by combining observations on seedlings from a greenhouse experiment (*N* = 8,199) and on adult tress in situ (*N* = 315). In the greenhouse, we followed half‐sib families for four growing seasons for growth and phenology traits, and tested their water stress response in a "drought until death" experiment. Adult trees in the field were assessed for *δ* ^13^C, a proxy for water use efficiency, and genotyped at 357 SNP loci. SNP data was used to generate a null expectation for seedling trait divergence between populations in order to detect the signature of selection, and 31 environmental variables were used to identify the selective environment. We found that seedlings originating from populations with low soil water capacity grew more slowly, attained a smaller stature, and resisted water stress for a longer period of time in the greenhouse. Additionally, adult trees of these populations exhibited a higher water use efficiency as evidenced by their *δ* ^13^C. These results suggest a correlated evolution of the growth‐drought tolerance trait complex. Population divergence in bud break phenology was adaptive only in the second growing season, and evolved independently from the growth‐drought tolerance trait complex. Adaptive divergence in bud break phenology was principally driven by the inter‐ and intra‐annual variation in temperature at the geographic origin of the population. Our results illustrate the different evolutionary strategies used by populations to cope with drought stress at the range limits across a highly heterogeneous landscape, and can be used to inform assisted migration programs.

## 1 INTRODUCTION

As a consequence of global warming, an increase in drought‐triggered tree mortality and vegetation shift has been reported worldwide (*e.g*. Allen et al., 2010; Allen, Breshears, & McDowell, 2015; Asner et al., 2016; Rigling et al., 2013). Progressively increased frequency and severity of drought events have caused defoliation (*e.g*. Carnicer et al., 2011) and large‐scale forest die‐off events (*e.g*. Breshears et al., 2005), and fostered the appearance of other detrimental agents such as insects (*e.g*. Anderegg et al., 2015). Damage due to drought may also be amplified by physical disturbances such as fires (*e.g*. Littell, Peterson, Riley, Liu, & Luce, 2016) or storms (*e.g*. Csilléry et al., 2017; Seidl et al., 2017). While die‐back events of adult trees are raising alarms due to their considerable effects on ecosystem functioning, carbon balance, and economy (Trumbore, Brando, & Hartmann, 2015), drought and soil desiccation are also primary limiting factors for the establishment and growth of seedlings (Moles & Westoby, 2004; Padilla & Pugnaire, 2007). Differential recruitment between species in response to drought stress could also alter forest structure and composition (e.g. Gazol, Camarero, Sangüesa‐Barreda, & Vicente‐Serrano, 2018; Herrera, 1992; Mendoza, Gómez‐Aparicio, Zamora, & Matías, 2009; Thrippleton, Bugmann, Folini, & Snell, 2018). Finally, extensive seedling mortality may lead to delayed forest decline, a phenomenon called "extinction debt" (Hanski & Ovaskainen, 2002; Talluto et al., 2017).

Mechanisms to reduce the negative impacts of drought encompass changes from the micro, cellular or organ level, such as stomatal closure, to macro scales, such as phenological shifts (Moran, Lauder, Musser, Stathos, & Shu, 2017). The role of different traits in resisting drought stress may change across life‐stages (Donohue, 2014; Juenger, 2013). Studies assessing drought tolerance in forest trees often reflect a dichotomy of research between small and large trees (McDowell, Ryan, Zeppel, & Tissue, 2013). Drought stress response of adult trees is most often assessed in tree rings using growth patterns (*e.g*. Vitali, Büntgen, & Bauhus, 2017) or stable isotopes (*e.g*. Penuelas, Hunt, Ogaya, & Jump, 2008). In contrast, studies of seedlings are generally performed in a controlled environment and compare growth patterns of mesic and xeric provenances (*e.g*. Rehfeldt, 1986; Kreyling et al., 2014), or assess the response of different populations to a water stress treatment (*e.g*. Warwell & Shaw, 2019), or use stable isotopes to understand the genetic architecture of drought stress response (*e.g*. Brendel, Pot, Plomion, Rozenberg, & Guehl, 2002). Experimental common garden studies of seedlings using half‐ or full‐sib families are particularly important for studying adaptation to drought stress, because they allow for the partitioning of the phenotypic variance into genetic and environmental components, thereby estimating the adaptive potential of traits or testing if trait divergence between populations suggests adaptation (Alberto et al., 2013; Whitlock, 2008).

It is commonly postulated that trees at the seedling stage experience the strongest selection pressure in nature, while adult trees are fairly resistant to abiotic stressors (*e.g*. Savolainen, Pyhäjärvi, & Knürr, 2007). Although selective survival of drought tolerant seedlings could be an efficient strategy to establish drought adapted populations (Kuparinen, Savolainen, & Schurr, 2010; Petit & Hampe, 2006), this is only possible if there are genetic correlations between relevant seedling and adult drought tolerance traits (Moran et al., 2017). Unfortunately, there is only limited and indirect evidence for such genetic correlations because complex ontogenetic and environmental factors contribute to differences in drought tolerance between life stages. For example, seedlings often have higher photosynthetic capacity per leaf mass (*e.g*. Thomas & Winner, 2002) and higher nitrogen but lower water‐use efficiency than adult trees (*e*.g. Mediavilla & Escudero, 2003; Donovan & Ehleringer, 1991). Drought avoidance strategies of adults and seedlings can also differ because they have access to different water resources due to differences in their rooting depth. For example, Cavender‐Bares and Bazzaz (2000) found that seedlings of *Quercus rubra* resisted drought by closing stomata early in the day, while mature trees avoided drought by accessing deeper water reserves thanks to their deeper rooting systems.

The development of genomic resources in species with long generation times, such as forest trees, opens new horizons in forest tree breeding (Grattapaglia et al., 2018) and also in evolutionary research. Notably, it may become possible in the near future to reliably estimate the genetic relationship between individuals in situ (Gienapp et al., 2017), which could allow for estimating genetic correlations between seedling and adult traits. In the meantime, combining common garden and genetic marker data can provide a means of obtaining robust inferences of locally adapted populations across life‐stages (*e.g*. de Villemereuil, Gaggiotti, Mouterde, & Till‐Bottraud, 2016). When phenotypes are assessed across different populations in a common environment, population genetic divergence between traits (*Q*_ST_) can be compared to an expected divergence between populations obtained from neutral genetic markers (*F*_ST_) (Leinonen, McCairns, O’hara, & Merilä, 2013; Whitlock & Guillaume, 2009). In the simplest test, *Q*_ST_ greater than *F*_ST_ can be interpreted as a signature of selection on the given trait. However, methods have been proposed that generalize and improve the *Q*_ST_/*F*_ST_ approach by accounting for hierarchical population structure and allowing for joint inferences on multiple traits (Ovaskainen, Karhunen, Zheng, Arias, & Merilä, 2011), or by detecting selection on traits as an over‐dispersion of genetic values among populations (Berg & Coop, 2014). A few recent studies of forest trees have used these novel methods, e.g. in black alder (*Alnus glutinosa*) (De Kort et al., 2016), in English yew (*Taxus baccata*) (Mayol et al., 2019), and in silver fir (*Abies alba*) (Csilléry et al., 2020).

Southern rear‐edge forest tree populations are particularly promising study systems and potentially valuable seed sources in the context of mitigating drought risks (*e.g*. Fady et al., 2016; Aitken & Bemmels, 2016). Species range limits are often characterized by small population sizes, high degrees of isolation and restricted gene flow (Petit & Hampe, 2006). Theory predicts that adaptation is prevented under such conditions, particularly due to maladaptive gene flow from central populations (Kirkpatrick & Barton, 1997), however, empirical evidence often suggests a lack of decreased abundance or fitness at range limits (Sexton, McIntyre, Angert, & Rice, 2009). Further, several common garden studies from the Northern Hemisphere report that seedlings from southern and drier provenances are more tolerant to drought than northern populations, e.g. in lodgepole pine (Isaac‐Renton et al., 2018; Montwé, Isaac‐Renton, Hamann, & Spiecker, 2016), in Douglas fir (Eilmann et al., 2013), in European beech (Kreyling et al., 2014; Robson, Sánchez‐Gómez, Cano, & Aranda, 2012; Rose, Leuschner, Köckemann, & Buschmann, 2009), or in cork oak (Ramírez‐Valiente et al., 2018; Ramírez‐Valiente, Sánchez‐Gómez, Aranda, & Valladares, 2010). Indeed, these populations have been exposed to a gradual warming climate since the last glacial maximum (LGM), thus they could have evolved greater drought tolerance than core populations (Aitken, Yeaman, Holliday, Wang, & Curtis‐McLane, 2008; Hampe & Jump, 2011; Hampe & Petit, 2005; de Lafontaine, Napier, Petit, & Hu, 2018). Finally, a high degree of heterogeneity with respect to the selective environment coupled with gene flow between populations could have also favoured rapid adaptation of populations at their range limits (Kawecki, 2008; de Lafontaine et al., 2018; Polechova, 2018).

Here, we study adaptation to drought stress in silver fir (*Abies alba* Mill.) from mountainous areas of its southern range limit in the Mediterranean Alps. Fossil data and dynamic global vegetation modelling congruently suggest the presence of silver fir in the Mediterranean Alps during the LGM (Liepelt et al., 2009; Ruosch et al., 2016; Terhürne‐Berson, Litt, & Cheddadi, 2004). The Mediterranean Alps also harbor a high degree of environmental heterogeneity: climatic conditions can rapidly change from hot, thermo‐Mediterranean to colder and moister mountain‐Mediterranean (Sagnard, Barberot, & Fady, 2002). Silver fir populations of the Mediterranean Alps have long attracted the interest of botanists and foresters due to their autochthonous origin and unique floristic composition, such as silver fir growing next to *Quercus ilex* (Barbero and Bono, 1970; Fady et al., 1999). To better understand their evolution and value for adaptive forestry, a large scale greenhouse experiment was established between 1995 and 1999 using open‐pollinated progenies from these populations. Several, potentially adaptive, phenotypic traits, including growth, phenology, and water stress response, were recorded. It is notoriously difficult to perform a controlled drought stress experiment due to the complex plant soil interactions. Since there was no a priori knowledge about the drought tolerance of these populations, a simple "drought until death" experiment was performed to screen a large number of seedlings from a large geographic region represented by several populations and families. Sagnard et al. (2002) analyzed part of the phenotypic traits from 15 stands to assess if populations clustered according to previously identified phytosociological and phytoecological groups (see more details in Appendix A). Roschanski et al. (2016) have also used some of the traits from four populations to provide complementary evidence for *F*_ST_ outlier tests (see more details in Appendix A).

In this study, we shall exploit the full phenotypic data set generated in the above experiment and combine it with newly generated genetic and stable isotope data. First, we will test the hypothesis whether population differentiation in seedling traits is the result of neutral or selective processes using a demographic null model, whose parameters are estimated from genetic marker data. The distinction between neutral and selective processes is particularly important in the study region that comprises many small, isolated populations, and even includes an island population (Corsica). This is because, in such settings, genetic drift could also lead to important trait differentiation between populations (Whitlock, 2008). Second, drought is a complex environmental stressor with complex plant responses that may influence multiple traits at a time (Juenger, 2013), thus a multi‐trait framework to detect adaptation is essential (Csilléry et al. 2020, Duputié, Massol, Chuine, Kirkpatrick, & Ronce, 2012; Sinervo & Svensson, 2002). Thus, we will test for correlated responses and trade‐offs across different suits of traits, notably between the amount and the timing of growth, as well as, between growth traits and the response to water stress. Third, we will explore a large range of environmental variables, including topography, soil and climate to identify potential drivers of adaptive trait divergence. Finally, we will compare seedling traits measured in the greenhouse with a proxy of water use efficiency, *δ* ^13^C, measured in situ on the adult trees, and ask if adult traits follow the same trends across populations as the seedlings.

## 2 MATERIAL AND METHODS

### 2.1 Study system and sampling

**Figure 1:**
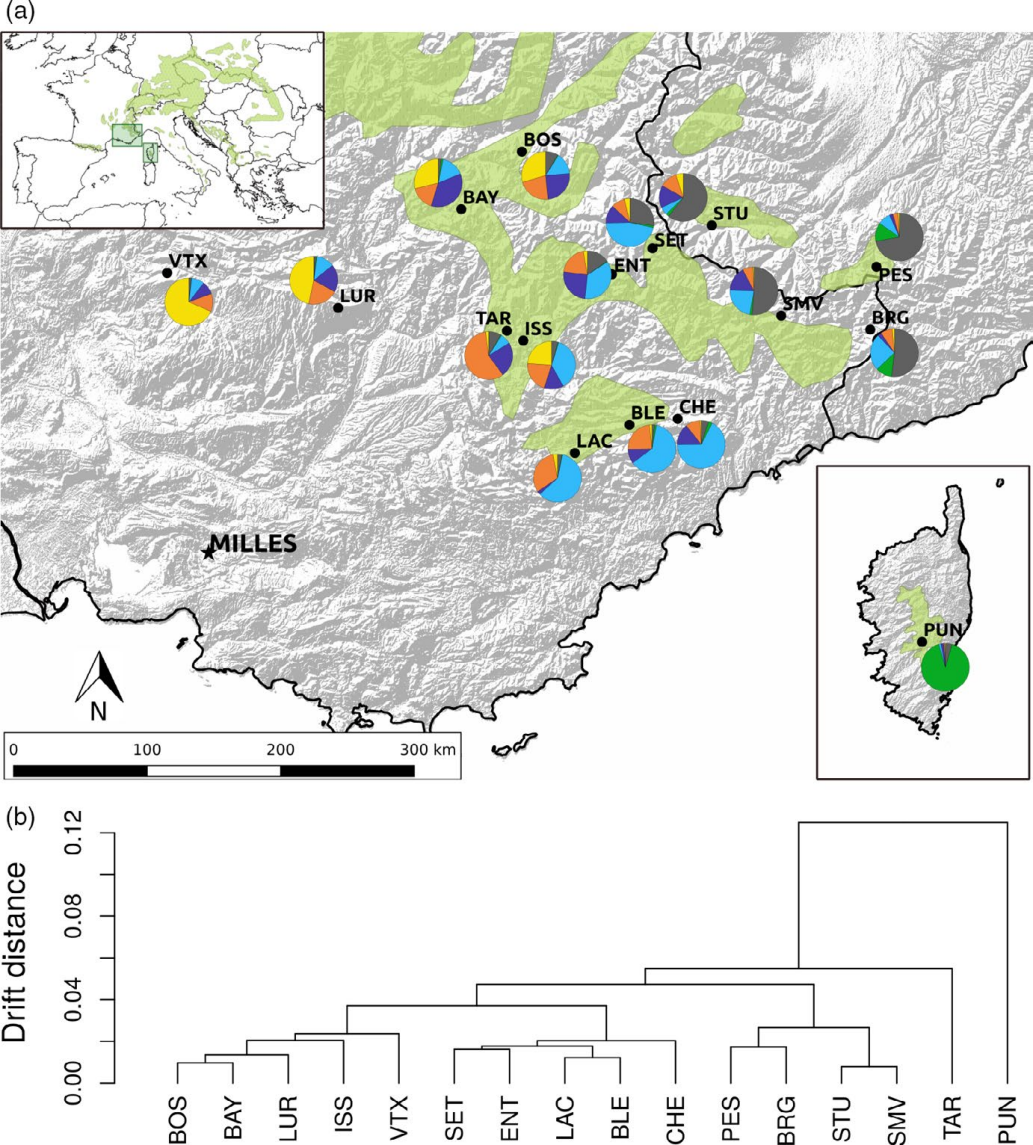
Population genetic structure of 16 silver fir (*Abies alba*) populations from the French Mediterranean Alps inferred from 357 SNP loci. (a) Geographic location of the sampling sites and proportion of individuals belonging to each of the six genetic clusters identified using the admixture model of the software *Structure*. (b) Neutral demographic, so‐called drift distances between populations estimated using the admixture F‐model implemented in the R package *RAFM*

We studied 16 silver fir stands across the Mediterranean Alps (Figure 1a, Table S1), subsequently referred to as populations. They were chosen to represent nearly natural populations based on the recommendations of the French National Forest Service (ONF) and the findings of Fady et al. (1999). Accordingly, all populations were autochthonous origin and managed using a close to nature forestry relying exclusively on natural regeneration. Populations were mostly pure *Abies alba* stands with less than 20% of other Mediterranean forest tree species, mainly *Fagus sylvatica*, *Picea abies*, *Pinus halapensis*, *Pinus nigra*, *Pinus sylvestris*, *Quercus ilex*, and *Quercus pubescens*. Five populations are classified as Genetic Conservation Units in the EUFGIS data base (http://portal.eufgis.org/), namely BOS, BRG, LUR, TAR, and VTX.

Populations were visited in 1994 for sampling seeds, and in 2016 for sampling needles. In the fall of 1994, seeds were collected from 18–43 dominant trees per population (mean: 29.5; see number of sampled trees per population in Table S1). These trees are subsequently referred to as mother trees and their offspring as families. Dominant trees were selected because they have larger crowns, more access to light and water resources, thus they generally produce more cones and seeds (Greene & Johnson, 1994). A minimum distance of 30 m was maintained between trees to avoid sampling close relatives. Isolated trees were also avoided. The weight of 1,000 seeds was recorded for each tree, and used as a proxy for maternal effects (St. Clair & Adams, 1991). In May 2016, within a period of three weeks, we sampled one year old, 2015‐grown needles from 20 trees at each site. At the time of sampling, the 2016 buds were elongated and, occasionally, the bud scales were broken. In a few cases, we were able to re‐identify the mother trees from 1994 and sampled these. Otherwise, we sampled other mature trees leaving a distance of at least 100 m between them.

### 2.2 Seedling traits from the greenhouse

In 1995, seeds were sown in an experimental forest nursery greenhouse located in Milles, near Aix‐en‐Provence in southeastern France (Figures 1, S1). Data from this greenhouse experiment has been partly described and part of the data have been analyzed by Sagnard et al. (2002) and by Roschanski et al. (2016) (see Appendix A for further details). The greenhouse was composed of an eastern and a western chamber, subsequently referred to as Greenhouse 1 and 2. Both greenhouses were divided into two experimental blocks, a northern and a southern block. Seedlings were grown in 600 cm^3^ containers, grouped in plastic crates by sets of 32 (Figure S1). Each mother tree was represented by 16–20 seedlings (mean: 17). Populations, families and seedlings were randomized across the four blocks with equal number of replicates in each. To account for border effects, seedlings were kept at a distance of approximately two meters from the greenhouse wall. In addition, extra rows of containers (identical to those used for testing) were placed at the edges of the experimental blocks, and were filled with a mixture of silver fir seedlings from the study area (Figure S1). A single row of containers were placed at the ends of blocks and five rows of containers along the sides of external blocks.

Forest nurseries are often located at lowlands and under more benign climatic conditions than the seed source sites for logistical reasons, but also to maximize seedling survival. In Milles, the mean annual temperature is 14.2°C and the mean annual precipitation is 555.5 mm, thus its climate is warmer and drier than that of the seed source sites (mean annual temperature of 6.2°C and mean annual precipitation of 1,163.8 mm across the 16 sites; see also Table S2), which is partly corrected by ventilation and watering. The ventilation of the greenhouse was assured by open doors at both ends. Additionally, shading walls were installed on the western, southern, and eastern sides of the greenhouse to reduce solar radiation and heat during the summer months. The northern side of the greenhouse was protected from the prevailing north‐western wind (Mistral) by a hedge (Figure S1). The need for watering across each block was estimated by continuously weighing one reference crate containing 32 seedlings, where several populations and families were mixed together. To eliminate drought stress, watering started at pF = 1.5, where pF is a measure of the soil water tension, thus the soil moisture was kept close to its field capacity (pF = 2). Field capacity is the water content of the soil after being watered and drained, i.e. when the moisture retention curve (or pF‐curve) is flat.

A common way of assessing drought stress tolerance under experimental conditions is using a "drought until death" treatment. Such treatment is destructive to the plants, thus no further traits can be assessed. In this experiment, we applied a "drought until death" treatment to all seedlings in Greenhouse 2. Starting from 6 May 1998, we completely stopped the watering in Greenhouse 2 in order to assess a single potentially adaptive trait: response to water stress. Greenhouse 1 continued to receive watering, which allowed us to continue scoring growth and phenology traits for an additional growing season. We stress that our aim was to compare the water stress response of different populations and families, and Greenhouse 1 did not serve as a control for the "drought until death" treatment. Performing the drought treatment in one greenhouse also assured a more uniform treatment because all seedlings had only non‐watered neighbours.

The aim of this experiment was to screen a large number of seedling across different populations and families, thus, we recorded traits that were simple to measure or observe. Each trait was recorded by at least two persons, and if the observations differed, their average was taken. Traits were recorded for three growing seasons starting from the 2nd (1997) to the 4th (1999) (Table 1). We analyzed observations on a total of 8,199 seedlings, of which 3,931 were in Greenhouse 1 and 4,267 in Greenhouse 2. Growth Increment and Height were used as raw measurements (trait names are capitalized hereafter; see Table 2 for trait definitions). Spring bud break phenology was scored at two dates each year from which we calculated a Bud Break Score as a sum of the two scores, each ranging from 1 to 5, with higher numbers indicated earlier bud break (see Table 2). Response to water stress was scored on five consecutive dates in 1998 (Table 1). At each date, a score between 0 and 3 was given, where low values indicate drought‐hardy seedlings and high values indicate drought sensitive seedlings. The sum of the five scores (raw Water Stress Score) was highly skewed and zero‐inflated (Figure S2). Thus, we calculated an integrative measure of water stress by weighting the stress scores with the log Julian dates of the observations. This weighted Water Stress Score had a close to Normal distribution (see Water Stress Score on Figure S2), and accounted for the evolution of the response to stress by giving higher weight to earlier responses to stress (i.e. to the most sensitive seedlings). The weighted Water Stress Score ranged between zero and 2.54.

**Table 1:** The time‐line of the experiment performed in the experimental forest nursery located in Milles (see Figure 1) and the number of observations recorded (N) in Greenhouse 1 and 2, respectively

Year	Period	Greenhouse	**Event**/Trait recorded	*N*
1995	Spring	1 and 2	**Sowing**	‐
1997	1 and 15 April	1 and 2	Bud Break Score	3,862 and 3,883
	Autumn	1 and 2	Growth Increment	3,208 and 3,620
	Autumn	1	Height	898
1998	3 and 17 April	1 and 2	Bud Break Score	1566 and 2,935
	6 May	2	**Stop watering**	‐
	5, 9, 12, 15, 17, and 22 June	2	Water Stress Score	3,620
	Autumn	1	Height	1683
1999	1 and 13 April	1	Bud Break Score	3,592
	Autumn	1	Height	3,705

Note

See Table 2 for definitions of the traits.

**Table 2:** Silver fir (*Abies alba* Mill.) seedling traits measured in the greenhouse experiment and their definitions

Trait	Definition
Bud Break Score	Sum of yearly scores recorded at two consecutive dates. The following scores were given at each date:
	1: Sleeping bud
	2: Swollen bud, scales are upright
	3: Needles are visible under the transparent scales
	4: Top of the needles broke through the bud
	5: Needles are completely free and grow
Growth Increment	Height difference between spring and fall in mm
Height	From soil to highest point in mm
Water Stress Score	Sum of scores recorded at five consecutive dates and weighed by the log Julien date. The following scores were given at each date:
	0: No visible sign of stress
	1: The side shoots point downwards
	2: The terminal shoot is not upright
	3: Needles are yellow across the whole plant

Not all traits have been measured in both greenhouses and for the three growing seasons. Hence, data collected on seedlings from Greenhouse 1 (*n* = 3,931) and Greenhouse 2 (*n* = 4,267) have been analysed separately as well as the data recorded across the three different growing seasons (but see Appendix C for some combined analysis). The sample size varied considerably among traits and years for various reasons (Table 1). First, the number of observations generally decreases with time for all traits due to mortality. Second, the 1997 Height measurement was missing for many seedlings because they grew only lateral branches, and could not be scored. Scoring additional traits reflecting the growth form was not feasible given the number of seedlings. Third, a late frost event hit most seedlings on 13 April 1998, which destroyed many terminal buds. This frost event impeded the scoring of bud break phenology in 1998, but also the lack of a leader shoot led to a bushy growth form, which also impeded many 1998 Height measurementswa

The response to the 13 April 1998 frost event was recorded as a binary response, thus not suitable for the following analysis of adaptive divergence (Karhunen, Ovaskainen, Herczeg, & Merilä, 2014). Nevertheless, we compared the frost damage between populations and blocks using a logistic regression model using the *glm* function in R with *family="binomial"*. We found a significant block effect on frost damage. The percentage of damaged seedlings were 38, 61, 35, and 56 across the blocks of Greenhouse 1 and 2, respectively, with the 61% (*p*‐value = .011) and 56% (*p*‐value = .043) blocks having significantly more damage in comparison to the block with 35%. In contrast, we found no population or block × population interaction effects (*p* > .112), thus it appeared sufficient to include block as a fixed effect in the following analysis to account for heterogeneity in the frost response.

### 2.3 Adult δ^13^C measured in‐situ

We used needles collected in 2016 to measure *δ* ^13^C, a proxy for water use efficiency, in 165 adult trees (on average, ten trees per population). Abaxial needles were selected to assure homogeneity of sampling among trees (Brendel, Handley, & Griffiths, 2003). The ten trees were selected to favour the widest possible spatial representation within each population, thus approximately 200 m between trees. This sampling assured that the environmental heterogeneity of the site that may influence *δ* ^13^C was represented and that between population variation reflected real population differences as opposed to local growing conditions of individual trees. Although *δ* ^13^C from wood cellulose is often preferred over needles for population studies, the two showed an extremely tight correlation in Scots pine (Brendel et al., 2003). Needles were stored in plastic bags at 5°C and were lyophiliozed for at least 48 hrs within a 20 days at most of their collection. *δ* ^13^C was measured at the ETH Grassland Sciences Isolab. Approximately 80 mg of lyophilized needle material was milled in 2 ml polypropylene tubes equipped with a glass ball (diameter of 5 mm) (4 min at 30 Hz). Milled samples were directly weighed into small tin capsules (approx. 5 mg, XPR2 microbalance from Mettler Toledo), and were combusted in an elemental analyzer (Flash EA by Thermo Finnigan, Bremen, Germany) coupled to an isotope ratio mass spectrometer (Delta XP by Thermo Finnigan, Bremen, Germany) by a Conflo II interface (Thermo Finnigan, Bremen, Germany). All carbon isotope values are reported on the Vienna‐Pee Dee Belemnite (V‐PDB) scale using the *δ*‐notation following Werner and Brand (2001).

### 2.4 Genotyping adult trees

We used needles collected in 2016 for extracting DNA and genotyping 20 trees per population, a total of 315 trees. We genotyped these at 377 single‐nucleotide polymorphism (SNP) loci using KASP arrays and the all‐inclusive service (i.e. DNA extraction and genotyping) from LGC Genomics (Middlesex, United Kingdom). We chose SNP markers due to their low mutation rate, which is an assumption of the method of Ovaskainen et al. (2011). Further, we attempted to select neutral SNPs, however, admittedly, this objective was difficult to fully achieve because the best genomic resource for the study species is the transcriptom of 12 trees (Roschanski et al., 2016). Nevertheless, due to the highly polygenic nature of most ecologically relevant traits (Csilléry, Rodríguez‐Verdugo, Rellstab, & Guillaume, 2018), it is extremely unlikely that we included any major quantitative trait loci, and we also excluded all putatively selective loci. We used loci that were developed by previous studies. First, we used 198 of the 267 SNPs from Roschanski et al. (2016): we excluded the SNPs that coded for non‐synonymous mutations and were detected as loci putatively under selection by previous studies (Brousseau et al., 2016; Heer et al., 2018; Mosca, González‐Martínez, & Neale, 2014; Roschanski et al., 2016). Second, we used 153 SNPs from the control panel of Mosca et al. (2012). Third, we used the 26 newly developed neutral loci from Csilléry et al. (2020). Genotypig success was 97.4% across the 377 SNPs. 20 loci were excluded, which had either a minor allele frequency less than 5% or more than 10% missing data, giving a total of 357 SNPs for further analysis.

### 2.5 Environmental data

Our aim was to identify putative environmental drivers of adaptation within our sampling area. We used downscaled historical climatic data to characterize environmental conditions at each sampling site. Our aim was to detect the signature of past selection on seedling traits. In order to obtain the closest representation of the selective environment that acted on past generations, we used data from 1 January 1901 to 31 December 1978. The choice of this period was justified by two facts: (a) no observation‐based climate data go back further in time, and (b) starting from approximately 1980, the temperature time series are overwhelmed by the effect of global warming (Harris, Jones, Osborn, & Lister, 2014). We used the delta method for statistical downscaling (Hay, Wilby, & Leavesley, 2000) to obtain mean monthly temperature and precipitation on a 1 km grid scale. The reference climatic data set for downscaling was the 0.5° resolution CRU TS v. 4.01 data (20 September 2017 release, Harris et al. (2014)), while the downscaling was based on the overlapping period with the 1 km resolution Chelsa data (Karger et al., 2017).

We calculated 31 variables to characterize the environment at each site (Table S2). Among potential geographic indexes, we only used longitude and latitude because elevation was highly correlated with some of the bioclimatic variables (Pearson correlation, *r* > 0.8, *p*‐value < .01), while slope was highly variable within sites. From the downscaled climate data, we calculated 19 standard bioclimatic variables (Booth, Nix, Busby, & Hutchinson, 2014) using the R package *dismo*, two potential evapotranspiration (PET) indexes and a standardized precipitation ‐ evapotranspiration index (SPEI, Beguería et al. 2014) using the R package *SPEI*, and two indicators of late frost (see Table S2). From the yearly time series of SPEI, we calculated indices of drought severity and frequency over the whole period of 78 years (see Table S2). Available water capacity (AWC) of the soil (volumetric fraction) until wilting point was obtained at a 250 m resolution from the SoilGrids250 data base for depth 5, 15, 30 and 60 cm (Hengl et al., 2017). 5 and 15 cm, and 30 and 60 cm were strongly correlated, so we averaged the correlated depths, and respectively called them awc10 and awc45 (see Table S2).

### 2.6 Statistical analysis

#### 2.6.1 Population structure and demography

We estimated the demographic distances between populations from SNP allele frequency variations. These distances can be used to derive a null expectation for trait divergence, and thereby to test for selection among populations in quantitative traits. We used the admixture F‐model (AFM), which assumes that populations diverged from a common ancestral pool and experienced only genetic drift (Karhunen & Ovaskainen, 2012). This model can disentangle small population size from isolation, which are the principal mechanisms underlying drift. We estimated the so‐called drift distances, i.e. the coancestry matrix, between all pairs of populations using a Metropolis‐Hastings algorithm implemented in the R package *RAFM* (Karhunen & Ovaskainen, 2012). We ran 10 independent Markov chains with a burn‐in of 20,000 iterations followed by 10,000 iterations for estimation with a thinning interval of 10, and averaged the posterior estimates of the coancestry matrix across chains. Additionally, we used the Bayesian clustering algorithm implemented in the software *Structure* v.2.3.4 (Falush, Stephens, & Pritchard, 2003; Hubisz, Falush, Stephens, & Pritchard, 2009; Pritchard, Stephens, & Donnelly, 2000) to find clusters of genetically related individuals across the sampled populations using the SNP data, thereby to validate the results obtained with RAFM (see Appendix B for details).

#### 2.6.2 Evolutionary potential and genetic correlations

We used the animal model (Henderson, 1975; Kruuk, 2004) implemented in R‐ASReml 3 (Butler, Cullis, Gilmour, & Gogel, 2009) to partition the trait variance into different components, and estimate the heritability and evolutionary potential of individual traits, and the genetic correlations between traits. We used the following statistical model for a single trait (univariate model):

$$y=Xb+Z_{p}p+Z_{m}m+Z_{a}a+e,$$

where **y** is a vector of observations for a trait on all seedlings, and **X** and **Z** are incidence matrices relating the fixed and random effects, respectively, to the observations. **b** indicates the fixed effect for block, **p** the random effect for population of origin, and **m** for seed weight, which was used as a proxy for maternal effects. **a** is a vector of individual breeding values with variance *Var*(**a**)=**A** × *V_A_*, where **A** is the relationship matrix estimated from the pedigree and *V_A_* is the additive genetic variance. Finally, *e* is the vector of residuals following *E*: *N* (0, *V_E_*), where *V_E_* is the error variance. Block was included as a fixed effect because it had only two or four values depending if one or both greenhouses were considered. We also performed multivariate models and estimated the additive genetic covariance between traits (*COV_A_*). In the multivariate models, **y** was a matrix of observations for multiple traits. We performed a thorough model selection and model checks for both uni‐ and multivariate models. Briefly, we tested the significance of all fixed and random effects, compared estimates using one or both greenhouses, compared the whole study area versus regional estimates of the variance components, tested the effect of the mating system on the variance components, and compared two, three and four trait multivariate models. The methods and results related to these analysis are provided in Appendix C.

We estimated the heritabilty from the univariate models as.

$$h^{2}=\frac{V_{A}}{V_{T}},$$

where *V_P_* is the total phenotypic variance (Falconer & Mackay, 1996), and the additive genetic coefficient of variation as

$$CV_{A}=\frac{V_{A}}{M},$$

where *M* is the trait mean (Houle, 1992). Finally, we estimated the genetic correlations between traits Falconer and Mackay (1996) as

$$r_{g}=\frac{COV_{A}}{\sqrt{V_{A1}\times V_{A2}}},$$

where *V_A_*_1_ and *V_A_*_2_ denote the additive genetic variance for trait 1 and 2, respectively.

#### 2.6.3 Adaptive divergence and environmental drivers

We used the test of adaptive divergence (*S*‐test) developed by Ovaskainen et al. (2011) implemented in the R package *driftsel* to distinguish between the effects of drift and natural selection in shaping trait values across the 16 populations (Karhunen & Ovaskainen, 2012). *driftsel* also implements an animal model similar to the above model, with the difference that it simultaneously accounts for the pedigree and the demographic distances between the populations estimated from genetic marker data (see above). To achieve this, *driftsel* co‐estimates the additive genetic mean and variance of the trait values in an assumed ancestral population, as well as the deviations from the ancestral mean for each population, i.e. the population effects (see further details in Ovaskainen et al. (2011)). Block was used as a fixed effect and seed weight as a covariate in the model in the same fashion as in the animal model above.

Ovaskainen et al. (2011) proposed the *S*‐test to summarize the evidence for adaptive divergence across all populations. *S* = 0 indicates consistency with homogenizing selection, *S* = 0.5 with neutrality, and with *S* = 1 with diversifying selection. In addition, and following Csilléry et al. (2020), we also summarized the evidence for adaptation in each population separately. Technically speaking, we will conclude that a particular population diverged more from the ancestral mean than expected by drift alone if 95% of the posterior distribution of the mean additive trait value is outside of the drift envelop. A modified version of the *driftsel* package implementing this population‐wise *S*‐test is available from Csilléry et al. (2020). Finally, note that *driftsel* uses a Bayesian approach, so we will use "unusual" and not "significant" to indicate results with strong evidence of adaptive divergence.

We ran three independent chains for each trait with a burn‐in of 100,000 iterations followed by 30,000 iterations for parameter estimation and using a thinning interval of 10. We ran separate analysis for the two greenhouses mainly because a Markov chain Monte Carlo analysis of *driftsel* was not computationally feasible for the full data set. Although *driftsel* can accommodate multivariate models, we were unable to reach convergence for trait‐pairs (tried up to 200,000 iterations, which involves a run time of over 4 weeks; results not shown). Thus, in order to summarize adaptive divergence in a multi‐trait space, we performed a Principal Component Analysis of the deviations of mean additive trait values from the estimated ancestral mean standardized by the expected trait variance based on drift. We used the *prcomp* function in R. This analysis combined the results from the 11 traits measured in the different greenhouses and years.

**Figure 2:**
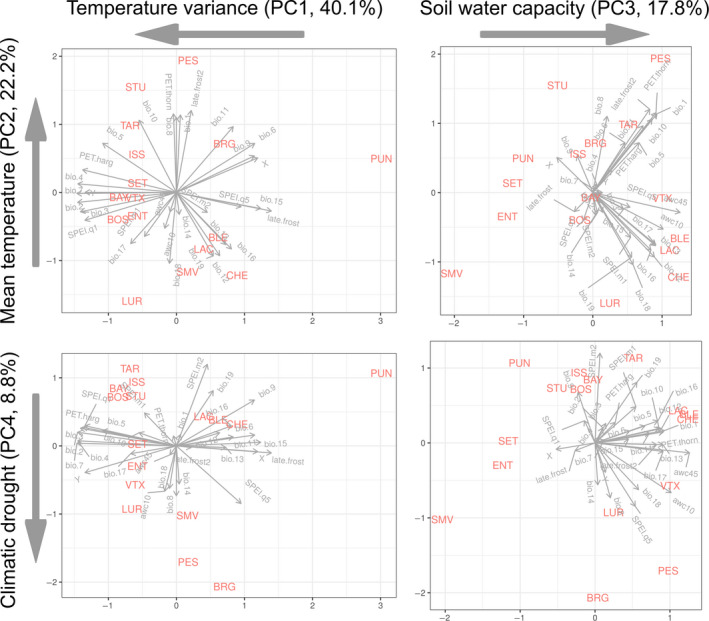
Situation of the 16 silver fir populations in a four dimensional environmental space defined by the first four Principal Components (PCs) of 31 environmental variables. Definitions of the environmental variables are given in Table S2 and their abbreviated names are marked on the grey arrows inside the plots. The first four PC axes explained 88.9% of the variance in the 31 environmental variables. Axis names indicate the synthetic names of the PC axes based on the environmental variables that had the highest loadings on them, and the variance that each axis explained. Loadings are given in Table S3. Arrows below the synthetic axis names indicate their interpretation from decreasing to increasing values

We used the *H* ^*^‐test to identify the potential environmental drivers of population divergence (Csilléry et al., 2020). Originally, the *H*‐statistic was proposed to measure whether the distance between the populations' mean additive trait value is more similar to environmental distances than expected based on drift alone (Karhunen et al., 2014). However, when *S* is close to one, *H* can also be close to one, even when selection is uncorrelated with the tested environmental driver. Csilléry et al. (2020) proposed a simple standardization to controls for such false positive outcomes (so that *H* becomes *H* ^*^). *H* ^*^ can be interpreted as a correlation coefficient: values close to one indicate that the degree of deviation from neutrality is correlated with the value of the environmental variable, thus as evidence for it being the driver of adaptation. The *H* ^*^ test is implemented in the modified *driftsel* package (Csilléry et al., 2020). To avoid multiple testing of the large number of environmental variables and enhance the interpretation of our findings, we reduced the environmental variables to synthetic variables using a Principal Component Analysis (PCA) implemented in the *prcomp* function in R. All variables were centered and scaled. The first four PC axes explained 88.9% of the variance (Figure 2). Based on the variables with the highest loadings on each of the PC axes (Table S3), the four PC axes will be referred to using synthetic names: Temperature variance (PC1), Mean temperature (PC2), Soil water capacity (PC3), Climatic drought (PC4) (Figure 2).

#### 2.6.4 Adult δ^13^C

First, we compared the adult δ*^13^C* between populations using a simple linear model implemented in the *lm* function in R. Further analysis was meaningful only given that part of the variation in *δ^13^C* is due to population of origin. Second, we correlated *δ^13^C* with the synthetic PC variables and with elevation to test what aspects of the environment influence this adult trait measured in situ. We also used elevation to test if δ13C increases with altitude, which is a consistent finding across different populations and taxa (Körner, Farquhar, & Wong, 1991). Finally, we correlated the mean *δ^13^C* with the mean additive genetic trait values of each population estimated with *driftsel*. Our aim was to test for consistencies in the ordering of populations with respect to adult and seedling trait values.

## 3 RESULTS

### 3.1 Population structure and demographic history

Using *Structure*, we identified six genetic clusters across the 16 silver fir populations. Not surprisingly, the population from the island of Corsica (PUN) was the most distinct from other populations (Figure 1a). The genetic structure of the mainland populations was characterized by isolation‐by‐distance both from east to west and from south to north (Figure 1a, see further details in Appendix B). Using *RAFM*, the variance between chains was nearly identical to the variance within chains (mean potential scale reduction factor (Gelman & Rubin, 1992) between 0.9 and 1.1 across ten chains), indicating a reliable estimation. With *RAFM*, PUN was again the most distant population, while the other populations were grouped into three clusters that corresponded to geographic regions of nearby populations possibly connected by gene flow (Figure 1b). The relative agreement between the two methods of inference is comforting because they have different assumptions: *Structure* clusters individuals to an arbitrary number of groups, while *RAFM* assume the presence of a single ancestral gene pool from which all populations had been derived while maintaining varying levels of gene flow.

### 3.2 Evolutionary potential and genetic correlations

**Figure 3:**
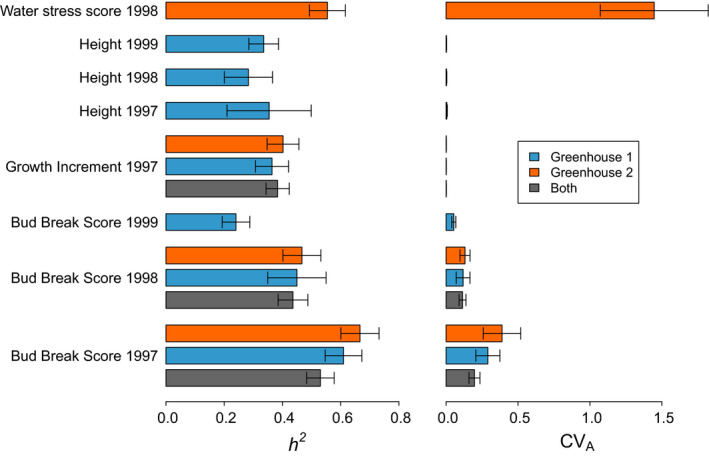
Heritability (*h* ^2^) and additive genetic coefficient of variation (*CV_A_*) of 11 traits measured in silver fir (*Abies alba*) seedlings in a greenhouse experiment involving two greenhouses and a total of 8,199 observations. Not all traits were scored in both greenhouses. Greenhouse 2 received a water stress treatment in 1998 after which only the Water Stress Score was recorded. Parameters were estimated using an animal model implemented in *ASreml‐R* including block as a fixed effect, and seed weight and population as random effects

All traits measured in the greenhouse experiment expressed a high heritability, with the largest value observed for Bud Break Score 1997, especially in Greenhouse 2 (0.69), while the lowest value, 0.24, was observed for Bud Break Score 1999 (Figure 3). The additive genetic coefficient of variation (*CV_A_*) was the highest for Water Stress Score, followed by Bud Break Score 1997, and it was negligible for Growth Increment and Height (Figure 3). Heritability and *CV_A_* estimates appeared robust whether using data from one or both greenhouses, across years and across regions of the study area (see Appendix C for more details). Multivariate analysis revealed several significant genetic correlations between pairs of traits. The strongest correlation was observed between Growth Increment 1997 and Water Stress Score 1998 (*r_g_* = 0.78; see also Figure A5 in Appendix C) indicating that seedlings from families that grew the least in 1997 were the most resistant to drought stress in 1998. The second largest, but negative, genetic correlation was observed between Height 1999 and Bud Break Score 1999 (*r_g_* = −0.64), indicating that seedlings from families that broke buds earlier reached a higher stature by the end of the same growing season. Growth‐related traits measured in consecutive years were also genetically correlated, e.g. between Growth Increment 1997 and Height 1999 *r_g_* was 0.55 (Figure A5 in Appendix C). Genetic correlations among all traits are given in Appendix C.

### 3.3 Adaptive trait divergence and its environmental drivers

**Figure 4:**
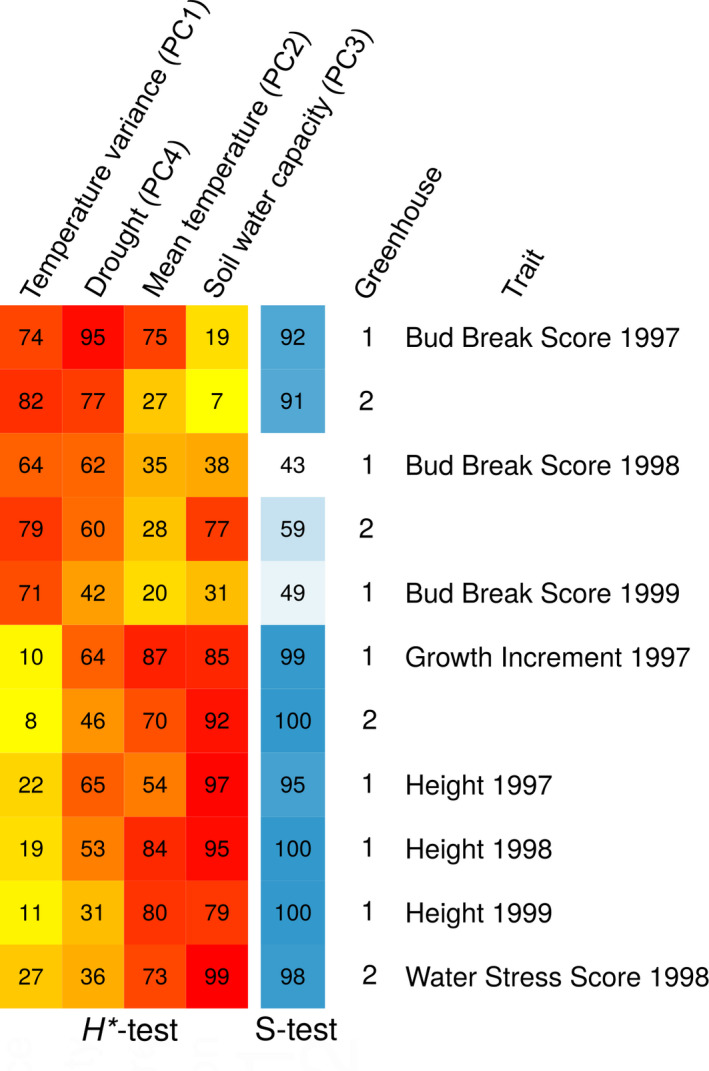
Evidence of adaptive divergence across 16 silver fir (*Abies alba*) populations at 11 traits measured on seedlings in a greenhouse experiment. The results of the *S*‐test of adaptive divergence (Csilléry et al., 2020; Ovaskainen et al., 2011) and *H* ^*^‐test of environmental drivers (Csilléry et al., 2020; Karhunen et al., 2014) are expressed as percentages. *S* close to 100% (in blue) indicate evidence for adaptation, while values close to 50% (in white) indicate neutrality. Values of *H* ^*^ can be interpreted as correlation coefficients, and indicate the role of the environment in driving adaptation. Strong evidence for the role of an environmental variable on a given trait is highlighted in red, while less relevant to irrelevant aspects of the environment are in shown from orange to yellow. *H* ^*^‐tests were performed on the synthetic environmental variables derived using a Principal Component Analysis (Figure 2)

We detected adaptive divergence using the *S*‐test in several traits (Figure 4). The *S*‐test relies on the assumption that trait divergence between populations, which cannot be explained by neutral demographic processes (i.e. drift) alone, is likely the result of adaptation. Similar to *RAFM*, satisfactory convergence was achieved with *driftsel* (mean potential scale reduction factor of 1.05 across three chains with a range between 0.85 and 1.15 across all traits). Overall, growth traits, i.e. Height and Growth Increment, and Water Stress Score showed stronger evidence of adaptive divergence (i.e. higher *S* values) than Bud Break Score (Figure 4). Spring bud break phenology showed adaptive divergence only in 1997, then the signal disappeared (i.e. *S*was close to 0.5 ). The environmental drivers of adaptation were also different among traits and showed a clustering into two distinct groups. While population divergence in growth traits and Water Stress Score was influenced by Mean temperature (PC2) and Soil water capacity (PC3), divergence in phenology was mainly influenced by Temperature variance (PC1), and Climatic drought (PC4) (*H* ^*^‐test, Figure 4 and 2).

**Figure 5:**
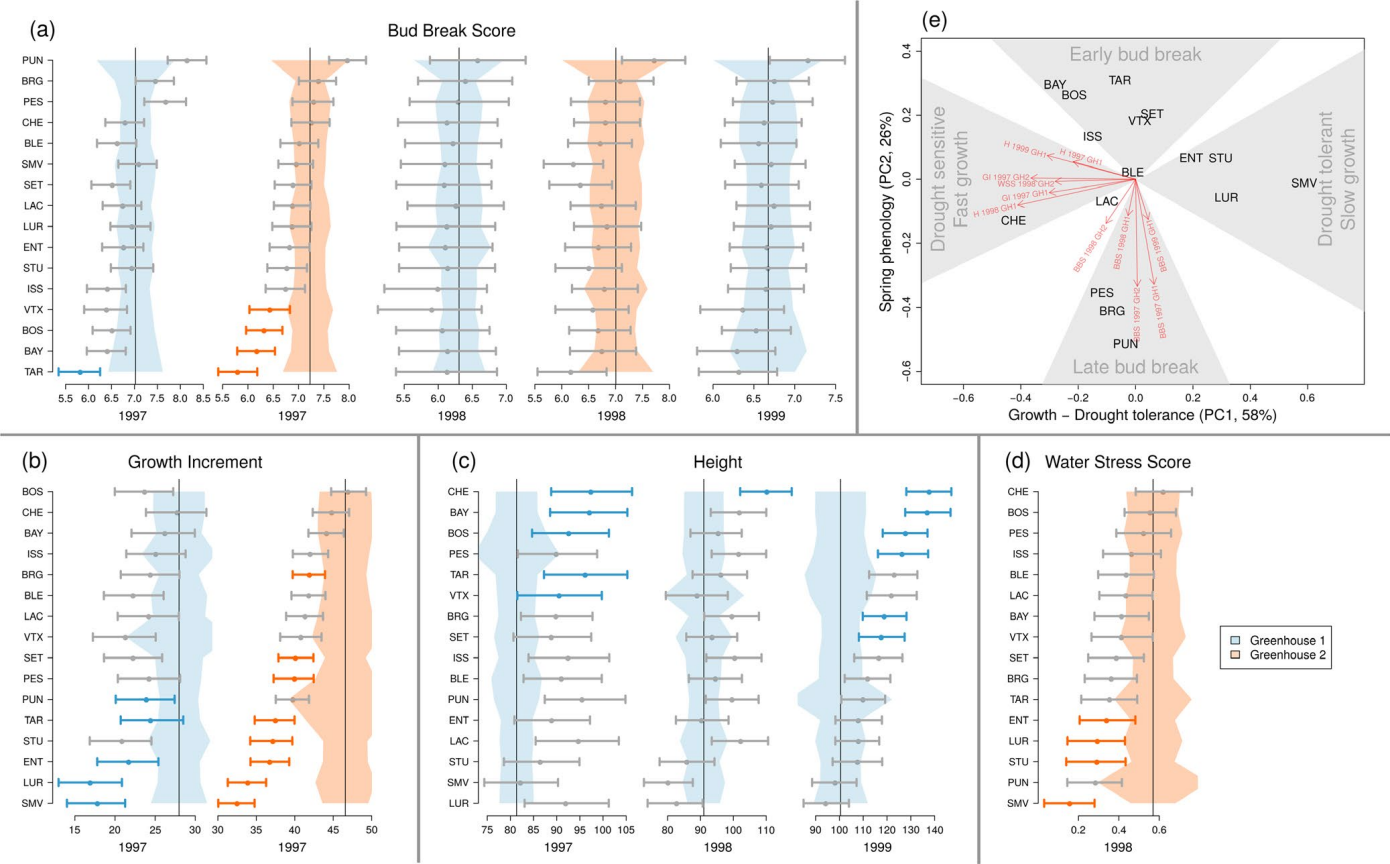
Evidence of adaptive divergence across 16 silver fir (*Abies alba*) populations at 11 traits measured on seedlings in a greenhouse experiment. Each trait is shown in a panel (a–d) that group together its values from different years and/or greenhouses. Each individual plot within panels shows the ancestral mean additive trait value (vertical line), the amount of trait divergence from this mean that is expected based on drift (light blue or orange envelop for Greenhouse 1 and 2, respectively), and the mean and 95% credible interval of the posterior distribution of the additive trait values for each population (horizontal error bars) estimated using *driftsel*. The horizontal error bars are blue or orange for populations with evidence for adaptive trait divergence; see Materials and Methods for details. Populations are ordered within each panel a‐d according to the trait values for the year/greenhouse with the highest number of unusually diverged populations. (e) Summary of divergence across all traits using a Principal Component Analysis (PCA); see Materials and Methods for details. The first two PC axes are given synthetic names (Growth‐Drought tolerance and Spring phenology) according to the variance they explained in the standardized trait divergence across the 11 traits

Next, using the population‐wise *S*‐test, we asked in which directions populations evolved from the hypothetical ancestral trait value. For this, we inferred ancestral trait values and the expected level of trait divergence based on drift alone, and contrasted these with the estimated mean additive genetic trait values of the populations (Figure 5a‐d). We detected unusually early bud break for TAR, BAY, BOS and VTX in 1997 (Figure 5a). These populations evolved under a typical continental environment, i.e. high variance in temperature, long drought periods, and winter precipitation in the form of snow (Figure 2). In contrast, PUN, BRG, PES had the latest bud break, but their additive genetic trait values were within a range that could be explained by neutral demographic processes alone (Figure 5a). Interestingly though, the climate at their geographic origin was the opposite of the populations with the earliest bud break, i.e. characterized by low temperature variance and lack of prolonged drought periods (Figure 2). This was particularly true for the Corsican island population (PUN), which had considerably less temperature variance than any other populations (Figure 2), yet due to its the large demographic distance from the mainland, the role of selection could not be concluded (Figure 5a).

Five to eight populations, depending on the greenhouse, showed unusually low Growth Increment: SMV, LUR, ENT, STU, TAR, PUN, PES, and SET (Figure 5b), and depending on the year, one to six populations exhibited unusually tall stature (i.e. Height): CHE, BAY, BOS, PES, TAR, VTX, BRG, and SET (Figure 5c). The fact that only one population showed unusual trait divergence for Height in 1998 could be due to the lower sample size in this year (see Table 1). The PCA analysis of the standardized trait divergences revealed that growth and height were strongly correlated (Figure 5e): populations that exhibited relatively fast growth also showed an unusually tall stature. Analysis of the environmental drivers suggested that higher soil water capacity (PC3) and warmer climate (PC2) led to enhanced growth (Figures 4 and 2).

Four populations had unusually high levels of drought tolerance, i.e. lower Water Stress Score: SMV, STU, LUR, ENT (Figure 5d). PUN had the second lowest Water Stress Score, but due to its large demographic distance from the mainland populations, we could not distinguish between drift and adaptation. We also found that drought tolerance and growth have likely evolved in a correlated manner: populations that evolved towards slower growth rates were more resistant to water stress, while populations that evolved towards a large stature were the least resistant to water stress (Figure 5e). These results are in agreement with the high genetic correlation between Growth Increment 1997 and Water Stress Score 1998 inferred using the standard animal model (see Appendix C). Furthermore, drought tolerance was influenced by a similar set of environmental variables as the growth traits (Figure 4). Finally, the PCA analysis of the standardized trait divergences suggested that spring phenology evolved independently from the growth‐drought tolerance trait complex (Figure 5e), which is not surprising, given that the two groups of traits also responded to different environmental cues (Figure 4).

### 3.4 ***Adult*** *δ* ***^13^C***

We found that populations of adult trees differed in their δ^13^ *C* (ANOVA, *df* = 15, *F* = 5.208, *p* < .0001, Figure S3), suggesting that ten individuals were sufficient to reveal population differences. Mean δ^13^ *C* was marginally significantly correlated with Mean temperature (PC2, Pearson's correlation, *r* = −0.46, *p*‐value = 0.058), moderately correlated with Temperature variance (PC1, *r* = 0.44, *p*‐value = .082) and Climatic drought (PC4, *r* = −0.35, *p*‐value = .180) and not related to Soil water capacity (PC3, *r* = −.21, *p*‐value = .432). δ^13^ *C* was strongly correlated with elevation (*r* = .82, *p*‐value < .001) and with raw variables, bio.5 (Max Temperature of Warmest Month) and bio.10 (Mean Temperature of Warmest Quarter), showing correlations of 0.71 and −0.68, and Bonferroni corrected p‐values of 0.011 and 0.018, respectively.

**Figure 6:**
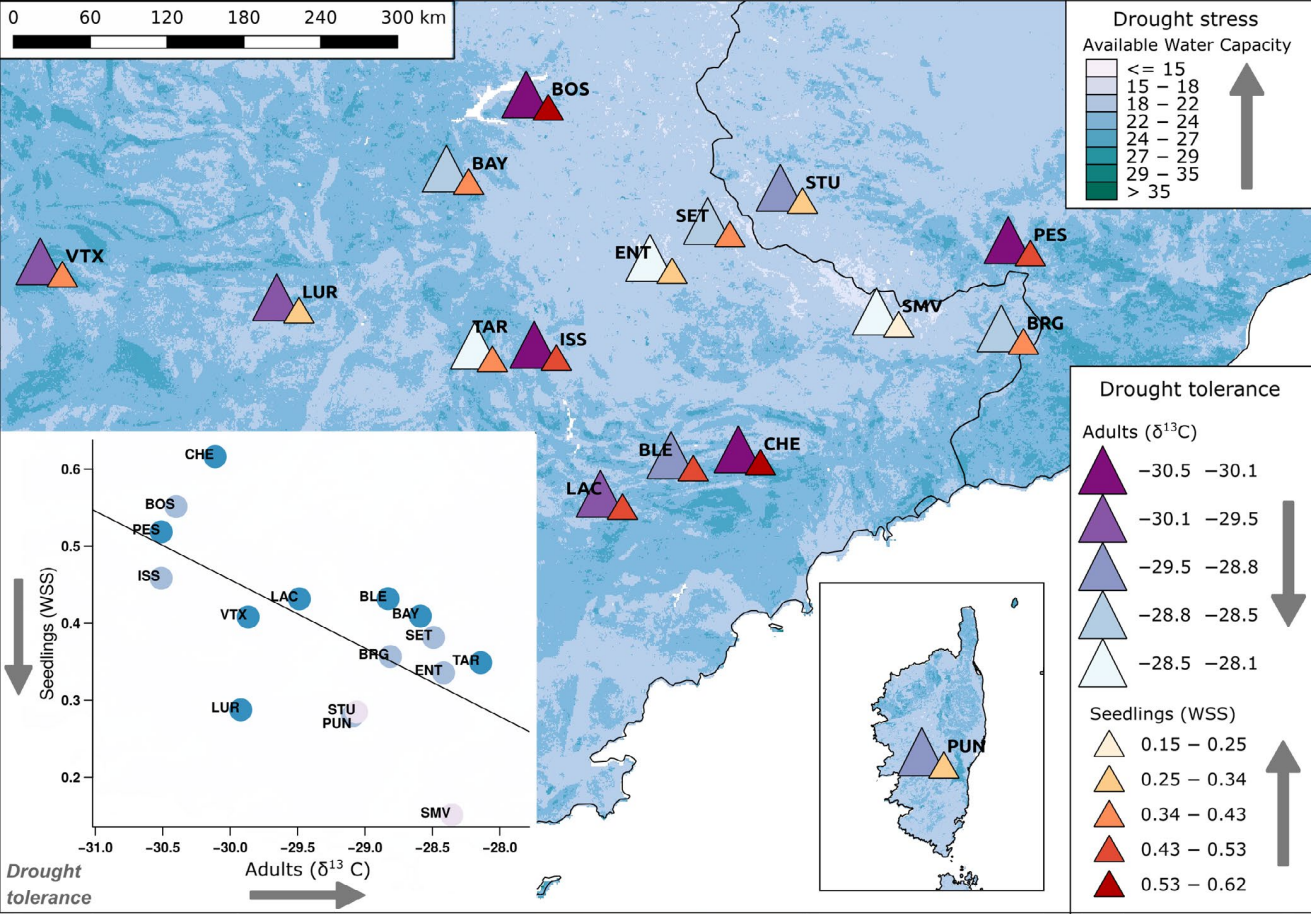
Adaptive landscape of drought tolerance across 16 silver fir (*Abies alba* Mill.) populations based on evidence from seedlings and adults (key entitled "Drought tolerance"). Each population is represented by two triangles, showing the estimated drought tolerance of the population based on evidence from adults (large triangles, δ^13^ *C*, measured in situ) and seedlings (small triangles, additive genetic trait values of Water Stress Score (WSS), measured in the greenhouse). The insert scatter plot shows the correlation between adult δ^13^ *C* and seedling WSS; points are color coded according to the Available Water Capacity at 30 cm depth (key entitled "Drought stress"), as well as the background of the map. Available Water Capacity at 30 cm was chosen because it had the highest loading on the synthetic variable Soil water capacity (PC3, Figure 2), which appears to be the main selective driver of the evolution of drought tolerance among the studied populations

Finally, we compared evidence for adaptive divergence in drought tolerance using drought stress response in seedlings and δ^13^ *C* in adult trees. We found that the populations' mean δ^13^ *C* was strongly correlated with seedling's mean additive trait value for Water Stress Score (*r* = −.638, *p*‐value = .008, Figure 6). As a result, adult trees of populations with a high water use efficiency had seedlings that were more resistant to the water stress treatment, i.e. had lower Water Stress Scores, in greenhouse conditions. The main environmental drivers of drought tolerance in seedlings were Soil water capacity (PC3) and, to a lesser extent, Mean temperature (PC2) (Figure 4, Table S3). A visual inspection of Figure 6 helps to understand this result: sites with low available water capacity tend to have more drought tolerant populations as indicated by adult trees' high δ^13^ *C* and seedlings' low Water Stress Score (i.e. "light colors go together" including the triangles and the background), even if considerable variation is present across the populations and the landscape.

## 4 DISCUSSION

### 4.1 Adaptive divergence in growth and drought stress response

We found evidence for adaptive divergence among silver fir populations from the southern limit of the species distribution range for growth and drought tolerance. In particular, we identified a trade‐off between these two suites of traits: seedlings originating from populations with relatively slow growth and small stature were the most drought tolerant both in terms of seedling traits measured in the greehouse and in terms of adult traits measured in situ (Figures 5 and 6). Genetic correlation between growth and drought tolerance has been demonstrated in several previous studies and across different life stages. For example, several forest breeding trials found a strong negative genetic correlation between growth or biomass traits and foliar δ^13^ *C* from seedlings or young adult trees (*e.g*. Baltunis, Martin, Huber, & Davis, 2008; Voltas, Serrano, Hernández, & Pemán, 2006; Voltas, Chambel, Prada, & Ferrio, 2008; Johnsen, Flanagan, Huber, & Major, 1999). Growth‐δ^13^ *C* trade‐off has also been reported at the between population level. For example, Cregg and Zhang (2001) compared 12 Scots pine provenances from the species range grown in a common garden, and found that seedlings from drier seed sources survived longer under drought and had a higher δ^13^ *C*, but were also smaller and allocated more biomass to roots. Csilléry et al. (2020) found that populations from dry inner‐Alpine valleys in Switzerland had a higher water use efficiency, and their progenies grew more slowly in a common garden. Furthermore, growth and/or social status (i.e. dominant or not) versus drought tolerance trade‐off has widely been reported at both between‐ and within‐population levels in adult trees (*e.g*. Ryan, Phillips, & Bond, 2006; Grote, Gessler, Hommel, Poschenrieder, & Priesack, 2016; Lauteri et al., 2004), and a recent review claimed that this is a world‐wide tendency (Bennett, McDowell, Allen, & Anderson‐Teixeira, 2015). The underlying physiological mechanisms can vary, however, most authors agree that taller trees have a higher physiological vulnerability to hydraulic stress. Water transport to the canopy involves higher cost and higher evaporative demand with increasing tree size, thus tall trees experience increased risk of xylem embolism because tension in the water column increases with height (Koch, Sillett, Jennings, & Davis, 2004; Ryan et al., 2006). Also, several authors suggested that the negative correlation between δ^13^ *C* and height is the result of compromised photosynthetic capacity in drought resistant trees (*e.g*. Baltunis et al., 2008; Lauteri, Scartazza, Guido, & Brugnoli, 1997; Benowicz, Guy, & El‐Kassaby, 2000; Sun et al., 1996).

The main environmental drivers of drought tolerance and growth were proxies for the water holding capacity of the soil and temperature (Figures 4 and 6). The fact that a soil property is one of the main drivers of selection seems plausible because soil is generally more stable than climate. Indeed, forest trees, including silver fir, have large effective population sizes and long generation times, so evolution is slow in comparison to climate fluctuations. Using forest inventory data, a recent study also suggested that soil is more important than climate in determining the distribution of several trees species across Switzerland, including that of silver fir (Walthert & Meier, 2017). The role of soil properties can be particularly relevant in climatically dry areas. For example, Chakraborty, Jandl, Kapeller, and Schueler (2019) studied 29 provenance trials using Norway spruce in Austria, and found that climate determined the growth of provenances originating from colder, high elevation sites, while for warmer, lower elevation sites, climate and soil were equally important. There is even more abundant evidence from the Mediterranean for the role of soil in seedling survival (Padilla & Pugnaire, 2007; Villar‐Salvador et al., 2012). Finally, it could be that there is a literature bias towards the role of climate in adaptation to drought, simply because harmonized gridded climate data have been available for longer than that for soil.

### 4.2 Adaptive divergence in spring phenology

We found that adaptive divergence in bud break (only in the 2nd growing season, Figure 5) was weaker than, and independent of the growth‐drought tolerance traits (Figure 5e). From an evolutionary perspective, such decoupling of growth and phenology could be advantageous. Since many aspects of drought tolerance are limited by tree size, which cannot be changed from year to year, phenology could buffer some of the between year climatic fluctuations, including drought. The main environmental drivers of trait divergence in bud break were temperature variance at different temporal scales (daily, seasonal, and among years) and climatic drought variables, such as the frequency and severity in potential evapotranspiration indexes (Figures 2 and 4), which also supports this hypothesis. Adjusting to year to year climatic fluctuations can be particularly important in the southern range limit, which is often hit by late frosts. Other common garden and provenance trial studies also found that year‐to‐year variation in growth patterns was adjusted to the drought season, thereby suggesting that genetic variation in phenological traits could play a role in an adaptive drought stress response (*e.g*. George et al., 2019; Keller et al., 2011). In the past decades, as a result of increasing temperatures and earlier springs, significant increases in the length of the growing season have been documented (*e.g*. Linderholm, 2006; Menzel et al., 2006). However, if there is also an decrease in the water supply, forest trees may not always be able to benefit from this longer growing season. For example, Eilmann, Zweifel, Buchmann, Graf Pannatier, and Rigling (2011) studied mature Scots pine trees in a dry inner‐Alpine valley using an irrigation experiment, and found that control trees, suffering from drought, significantly shortened their actual growth period to a much shorter period than the phenological growth period.

The signature of adaptive divergence for spring phenology was no longer present in the 3rd and 4th growing seasons (Figures 4 and 5a). It is likely that the spatial scale of the study was not sufficient to demonstrate strong population genetic divergence in phenology. Spring phenology is generally determined by a combination of three key climatic factors, chilling, photoperiod and temperature, among which photoperiod has the strongest genetic component (Körner & Basler, 2010). Within the limited latitudinal range of the studied populations, the photoperiod can be considered constant, thus little genetic variation is expected for bud break phenology. Nevertheless, it is also possible that the difference between the temperature at the seed source sites and the greenhouse (8°C, on average) had a homogenizing effect across populations. Alternatively, unmeasured maternal and environmental effects specific to different populations can inflate estimates of the population genetic differentiation (*e.g*. Merilä & Crnokrak, 2001). Maternal effects are often stronger on earlier than later traits, which could explain the signal of adaptive divergence on Bud Break Score in 1997, but not in 1998 and 1999. Indeed, we also observed that seed weight, our proxy for maternal effects, explained a significant part of the trait variation in Bud Break Score only in 1997 and partly in 1998 (see Appendix C). Previous studies of silver fir have also documented a decrease in heritability and population genetic differentiation in phenology traits with years (*e.g*. Csilléry et al., 2020; Frank et al., 2017; Vitasse, Delzon, Bresson, Michalet, & Kremer, 2009), but such trends are not always consistent across other tree species (*e.g*. Hannerz, Sonesson, & Ekberg, 1999; Howe et al., 2003; Frank et al., 2017). Finally, we note that we did not measure traits that reflect the autumn phenology, while there is overwhelming evidence of adaptive clines for bud set (a proxy for growth cessation) in many forest tree species (*e.g*. Alberto et al., 2013). However, it appears that due to the deterministic bud development of *Abies* species, growth cessation is entirely determined by the spring phenology and previous year's vegetative season, thus it is not an adaptive trait for this genus (Cooke, Eriksson, & Junttila, 2012; Csilléry et al., 2020).

## 5 LIMITATIONS OF THE STUDY

Growing plants in containers of 600 cm^3^ was a potential source of stress for the seedlings, whcih may have altered their phenotypes in comparison to natural conditions. For example, containers may have limited the development of the root system, which could have influenced their growth and response to water stress. In particular, taller seedlings may have suffered more during the water stress treatment due to their higher rate of transpiration. Nevertheless, note that the evidence for populations being, on average, fast or slow growing and drought tolerant or sensitive were based independent observations: while Water Stress Score was evaluated in Greenhouse 2, most growth traits were scored in Greenhouse 1. Furthermore, taller trees could have changed their vessel size as a reaction to drought stress; a phenomena that has been observed in Scots pine (Eilmann, Zweifel, Buchmann, Fonti, & Rigling, 2009). Finally, it is also likely that the differences in stature at the onset of the water stress treatment had already reflected differences in drought tolerance. Indeed, the genetic correlation between growth and water stress response (0.78, see Appendix C) was considerably higher than the equivalent phenotypic correlation (Mean = 0.47, Range = 0.35–0.62 across populations), which suggests a strong character integration between growth and water stress response at the genetic level. However, this correlation does not allow for a causal interpretation concerning the relationship between the two traits and their respective developmental constraints.

The temperature of the greenhouse was hotter and more stable than the climates at the seed origin. The homogenizing nature of the experiment removed much of the environmental variance, which often results in inflated heritabilities in comparison to in‐situ values. For this reason, the absolute values of the quantitative genetic parameter reported in Figure 3 and in Appendix C should be interpreted only within the context for this experiment. As an example, a recent transplant experiment also using silver fir from the French Mediterranean Alps found that a much lower proportion of the trait variance was due to genetic factors than in this study (Latreille & Pichot, 2017). In contrast, heterogeneity across the different blocks was also an inherent feature of this experiment. The southern blocks had more frost damage because the openings on the southern side were not protected with a hedge like on the north side (Figure S1). Although we did not find evidence for the different populations being more or less sensitive to frost, admittedly, our evaluation of the frost damage was a simple one. Further, the westerly exposed Greenhouse 2 received more sun and was warmer. Indeed, the mean additive trait values across all populations were different between the two greenhouses in 1997, and seedlings grew less, on average, in the "colder" Greenhouse 1 (Figure 5). Nevertheless, the populations had a similar ordering in terms of additive trait values and similar degrees of divergence, thus there was no population × block interaction effect.

Silver fir is a predominantly outcrossing species, thus we assumed that all seedlings from the same mother tree are half‐sibs. However, the mating system in silver fir is likely more complicated, and composed of a mixture of outcrossing, bi‐parental inbreeding and selfing (Fady & Westfall, 1997). The degree of deviation from outcossing is influenced by a number of ecological factors such as population density. Restoux et al. (2008) found that outcrossing rate and variation can be the highest in low density populations and may vary from 0.43 to 0.87 in silver fir. The mating system can have a considerable effect on quantitative genetic parameters (Charlesworth & Charlesworth, 1995). In Appendix C, we show that the evolutionary potential would be three to four times lower if we assumed that all seedlings were issued from selfing.

## 6 CONSERVATION AND FOREST MANAGEMENT IMPLICATIONS

Although there is abundant evidence of past evolutionary responses of plants to climate, the pace of ongoing climate change is much faster than it has been during the time since post‐glacial expansion/re‐colonization (Stocker et al., 2014). Whether adaptive evolution can compensate for the effects of ongoing human‐induced climate change is unclear. In the case of silver fir, die‐back events have been documented in the study area (Cailleret, Nourtier, Amm, Durand‐Gillmann, & Davi, 2014), and reduced growth patterns have been reported in other rear‐edge populations in south‐western Europe (Gazol et al., 2015). Silver fir may be able to survive in the region by adapting to a warmer and dryer climate (Stocker et al., 2014), so‐called evolutionary rescue (Bell, 2017), but it could also be outcompeted by other more drought tolerant species. It has been speculated that late successional tree species such as silver fir may lag behind early successional species in tracking their respective climatic niches (Corlett & Westcott, 2013), but the opposite has also been suggested (Dyderski, Paź, Frelich, & Jagodziński, 2018). Given these these model uncertainties, it increasingly accepted that to mitigate climate change, active management may be necessary, especially for foundation and resource‐production species, such as forest trees (Aitken & Bemmels, 2016). The two possibilities are breeding for drought tolerance and assisted migration. Below, we discuss these two options in the light of our results.

Forest breeding practices have long been selecting trees for increased productivity based on growth‐climate relationships (Leites, Rehfeldt, Robinson, Crookston, & Jaquish, 2012). However, forest tree breeding programs are increasingly including selection criteria related to drought tolerance to meet the challenge of climate change, often using δ^13^ *C* as a target trait (*e.g*. Marguerit et al., 2014; Samuelson, Johnsen, Stokes, Anderson, & Nelson, 2018; Moran et al., 2017). Here, we found a high heritability and evolutionary potential for water stress response across Mediterranean silver fir populations, which suggests that rapid adaptation to elevated drought conditions may be possible (Figure 3). However, our study demonstrated that adaptive divergence in growth and water stress responses are correlated, thus the evolution of drought tolerance is linked to that of growth (Figure 6e). It has been suggested before that a negative genetic correlation between δ^13^ *C* and growth could compromise breeding results for productivity (Samuelson et al. 2018). We believe that it is unlikely that the evolution of the two traits could be decoupled due to the physiological and biophysical limitations discussed above. Indeed, for example, Lamy et al. (2014) found little plasticity for cavitation resistance, the ability to conduct water through the xylem during drought events. Although Domec et al. (2008) found that structural changes of the xylem in Douglas fir could satisfy hydraulic requirements for tall stature, such changes imposed increasing constraints on water transport efficiency. In conclusion, genetic improvement for both growth and drought tolerance can be possible, but only to an extent that ultimately depends on the genetic architecture of the traits (*e.g*. Resende et al., 2012).

Translating studies of local adaptation to management practice is a challenging and multi‐faceted task (Gibson, Espeland, Wagner, & Nelson, 2016; Sang, Sebastian‐Azcona, Hamann, Menzel, & Hacke, 2019). Differences between the adaptive strategies of the 16 Mediterranean silver fir populations could contribute to defining assisted gene flow strategies within the study area. Monitoring recruitment in situ and measuring water use efficiency in additional populations along a soil water holding capacity gradient could provide further evidence for adaptive forestry in the region. Although, it is likely that drought tolerance in all 16 populations is high in comparison to populations in the northern and more mesic part of the distribution range, Csilléry et al. (2020) found that dry inner‐Alpine valleys in Switzerland had a δ^13^ *C* that is comparable to that of populations studied herein. Finally we stress that planting southern provenances at higher latitudes to mitigate climate change is not always the optimal solution. Southern populations adapted to arid habitatscan be at a competitive disadvantage in more mesic environments because of hydraulic and physiological constraints and shorter day length (*e.g*. Eilmann et al., 2011; Liepe et al., 2016, Gessler, Schaub, & McDowell, 2017) .

## CONFLICT OF INTEREST

None declared.

## Supporting information

Supplementary Material

## Data Availability

Data used in the study is available at the Dryad Digital Repository https://doi.org/10.5061/dryad.f4qrfj6t3.
