# Supplementary material for: Adaptation to drought is coupled with slow growth, but independent from phenology in marginal silver fir (Abies alba Mill.) populations
Source: Evol Appl. 2020 Jun 17;13(9):2357–76. doi: 10.1111/eva.13029 (PMC7539328; doi:10.1111/eva.13029)
Supplement: Supplementary file 1 — Supplementary Material [file EVA-13-2357-s001.pdf]

Supporting Information

Title: Adaptation to drought is coupled with slow growth, but independent from phenology in marginal silver fir (*Abies alba* Mill.) populations

The following Supporting Information is available for this article:
Table S1 Names, political and geographic situation of the 16 silver fir (*Abies alba* Mill.) populations.
Table S2 Summary of the 31 geography and environmental variables characterizing the 16 populations.
Table S3 Loadings from Principal Component Analysis (PCA) of 31 environmental variables.
Figure S1 Photos and schema of the greenhouse experiment.
Figure S2 Raw data: distribution and correlation between seedling traits measured in the greenhouse experiment.
Figure S3 Raw data: δ^{13}C across the 16 populations.
Appendix A Use of the greenhouse phenotype data in Sagnard et al. (2002), Roschanski et al. (2016) and in this study
Appendix B: Inference of population structure using the Bayesian clustering algorithm implemented in the software *Structure*
Appendix C Estimating variance components using an 'animal model': model specification and tests of robustness.

Table S1: Abbreviated names of the 16 silver fir (*Abies alba* Mill.) population used throughout the paper, the full names of the sampled forests, and their political and geographic situation. Elevation is given in meters, and corresponds to the average elevation of the sampled mother trees. N indicates the number of mother trees whose progenies have been analysed for each population.

Abbreviation	Forest	Department	Country	Longitude	Latitude	Elevation	N
BAY	Forêt domaniale des Gorges-Du-Sasse	Alpes-de-Haute-Provence	France	6.25	44.35	1370	28
BLE	Forêt domaniale de Bleyne	Alpes-maritimes	France	6.83	43.82	1500	43
BOS	Forêt domaniale de Boscodon	Hautes-Alpes	France	6.46	44.49	1440	33
BRG	Forêt communale de la Brigue	Alpes-maritimes	France	7.65	44.05	1550	38
CHE	Forêt domaniale de Cheyron	Alpes-maritimes	France	6.99	43.84	1270	31
ENT	Forêt communale d'Entraunes	Alpes-maritimes	France	6.77	44.19	1590	26
ISS	Forêt domaniale de l'Issole	Alpes-de-Haute-Provence	France	6.46	44.03	1255	37
LAC	Forêt communale de la Bastide	Var	France	6.64	43.75	1295	31
LUR	Forêt communale de Cruis	Alpes-de-Haute-Provence	France	5.83	44.11	1340	19
PES	Alta Valle Pesio e Tanaro	Piedmont	Italie	7.67	44.21	1200	19
PUN	Forêt regionale de Punteniello	Corse	France	9.12	41.99	1600	41
SET	Forêt communale de Saint-Etienne-De-Tinée	Alpes-maritimes	France	6.90	44.25	1500	26
SMV	Forêt communale de Saint-Martin-Vesubie	Alpes-maritimes	France	7.34	44.09	1600	32
STU	Valle Stura	Piedmont	Italie	7.11	44.31	1100	18
TAR	Forêt communale de Tartonne	Alpes-de-Haute-Provence	France	6.41	44.05	1350	26
VTX	Forêt communale de Beaumont-Du-Ventoux	Vaucluse	France	5.15	42.12	1220	24

Table S2: Geography and environmental variables calculated for the period of 1 January 1901 - 31 December 1978 from monthly mean, minimum and maximum temperature and total precipitation of the CRU TS v. 4.01 data (Harris et al., 2014) downscaled using Chelsa data (Karger et al., 2017). Soil variables were extracted from the SoilGrids250 data base (Hengl et al., 2017). Abbreviations: PET: Potential Evapotranspitation; SPEI: Standardised Precipitation-Evapotranspiration Index, AWC: Available Water Capacity.

Variable	Description	Mean	(Min., Max.)
**Geography**			
Long	Longitude (degrees)	6.9	$(5.2, 9.1)$
Lat	Latitude (degrees)	44	$(42, 44.5)$
**Standard bioclimatic indexes**			
bio.1	Annual Mean Temperature	6.2	$(3.8, 8.2)$
bio.2	Mean Diurnal Range (Mean of monthly Tmax - Tmin))	8.4	$(5, 9.5)$
bio.3	Isothermality (bio.2/bio.7) (* 100)	25.1	$(19.6, 26.9)$
bio.4	Temperature Seasonality (standard deviation *100)	611.2	$(508, 649.3)$
bio.5	Max Temperature of Warmest Month	23.1	$(19.6, 25.2)$
bio.6	Min Temperature of Coldest Month	-10.3	$(-12.5, -5.8)$
bio.7	Temperature Annual Range (bio.5-bio.6)	33.5	$(25.3, 35.8)$
bio.8	Mean Temperature of Wettest Quarter	2.1	$(0, 6)$
bio.9	Mean Temperature of Driest Quarter	1.8	$(-2.8, 14.4)$
bio.10	Mean Temperature of Warmest Quarter	16.3	$(13.7, 18.3)$
bio.11	Mean Temperature of Coldest Quarter	-4.2	$(-6.4, -2)$
bio.12	Annual Precipitation	1163.8	$(801.3, 1671.9)$
bio.13	Precipitation of Wettest Month	396.5	$(271.6, 670.9)$
bio.14	Precipitation of Driest Month	0.3	$(0, 1.6)$
bio.15	Precipitation Seasonality (Coefficient of Variation)	66.6	$(60.6, 77.8)$
bio.16	Precipitation of Wettest Quarter	832.3	$(493.9, 1391.2)$
bio.17	Precipitation of Driest Quarter	35.7	$(11.2, 47.4)$
bio.18	Precipitation of Warmest Quarter	147.1	$(103.8, 204.5)$
bio.19	Precipitation of Coldest Quarter	367	$(107.6, 617.9)$
**Drought**			
PET.thorn	Mean annual PET (Thornthwaite)	43.2	$(37.4, 48.1)$
PET.harg	Mean annual PET (Hargreaves)	52.1	$(34, 59.4)$
SPEI.m1	Number of month with SPEI < -1	147.9	$(142, 153)$
SPEI.m2	Number of month with SPEI < -2	17.4	$(15, 19.2)$
SPEI.q5	5% quantile of SPEI	-1.6	$(-1.7, -1.6)$
SPEI.q1	1% quantile of SPEI	-2.2	$(-2.4, -2.1)$
**Late frost**			
late.frost1	Min temperature of the first month of the year with mean temperature > 5℃	1.8	$(1.2, 3.2)$
late.frost2	Min temperature of May	4.2	$(2, 6.7)$
**Soil**			
awc10	AWC (5-15cm)	49.7	$(44, 53)$
awc45	AWC (30-60cm)	43.6	$(35, 49)$

Table S3: Principal Component Analysis (PCA) of 31 environmental variables listed in Table S2. PC axes 1 to 4 explained 88.9% of the variance in the raw environmental variables. Column names show the synthetic names for the PC axes used in the paper, and the variance explained by each. The first ten environmental variables with the highest loadings are shown for each PC axes.

Temperature variance PC1 (40.1%)		Mean temperature PC2 (22.2%)		Soil water capacity PC3 (17.8%)		Climatic drought PC4 (8.8%)	
Variables	Loadings	Variables	Loadings	Variables	Loadings	Variables	Loadings
bio.7	-0.28	late.frost2	0.31	awc45	0.36	SPEI.m2	0.49
bio.2	-0.28	PET.thorn	0.3	awc10	0.29	SPEI.q5	-0.35
bio.4	-0.28	bio.1	0.29	bio.1	0.27	bio.19	0.33
bio.3	-0.27	bio.8	0.28	PET.thorn	0.26	bio.8	-0.3
late.frost	0.27	bio.10	0.27	bio.17	0.26	bio.9	0.28
PET.harg	-0.26	bio.18	-0.27	bio.13	0.26	awc10	-0.27
SPEI.q1	-0.26	bio.11	0.25	bio.12	0.26	bio.18	-0.26
Y	-0.26	bio.19	-0.24	bio.10	0.25	bio.14	-0.23
bio.15	0.24	bio.12	-0.24	bio.16	0.24	SPEI.m1	0.2
X	0.23	bio.16	-0.21	late.frost2	0.24	Y	-0.16

Supplementary figures

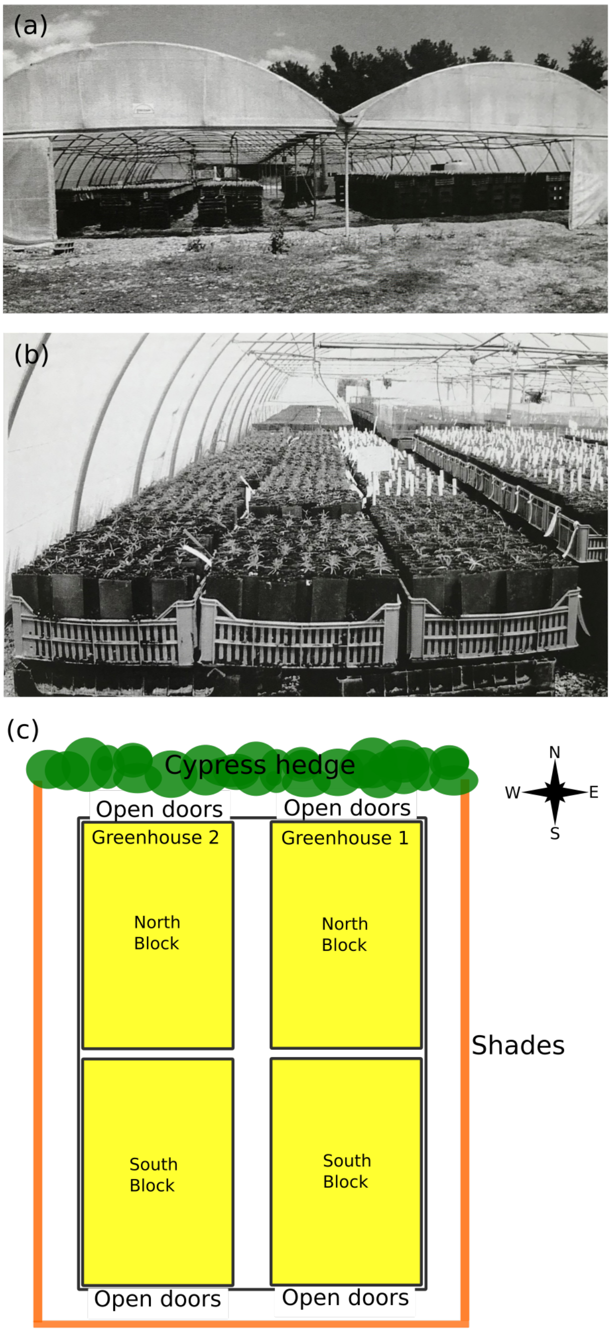

Figure S1: (a) View of the two greenhouse chambers (Greenhouse 1 and 2) from the south. (b) Rows of non-experimental *Abies alba* seedlings at the borders of the greenhouse to control for edge effects. Experimental seedlings were labelled with the white tags. (c) Schematic view and orientation of the experiment.

(a)

Greenhouse 1

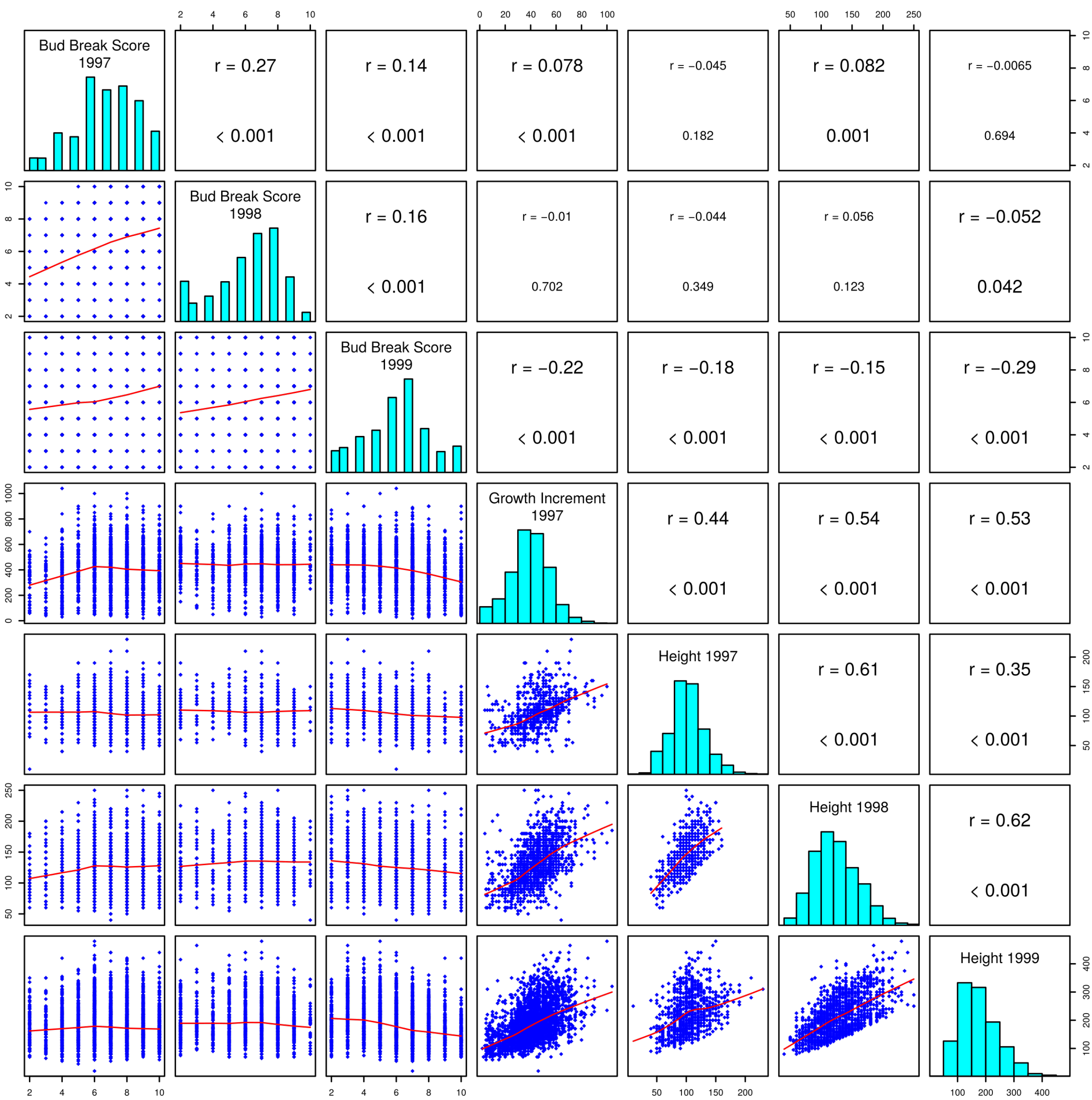

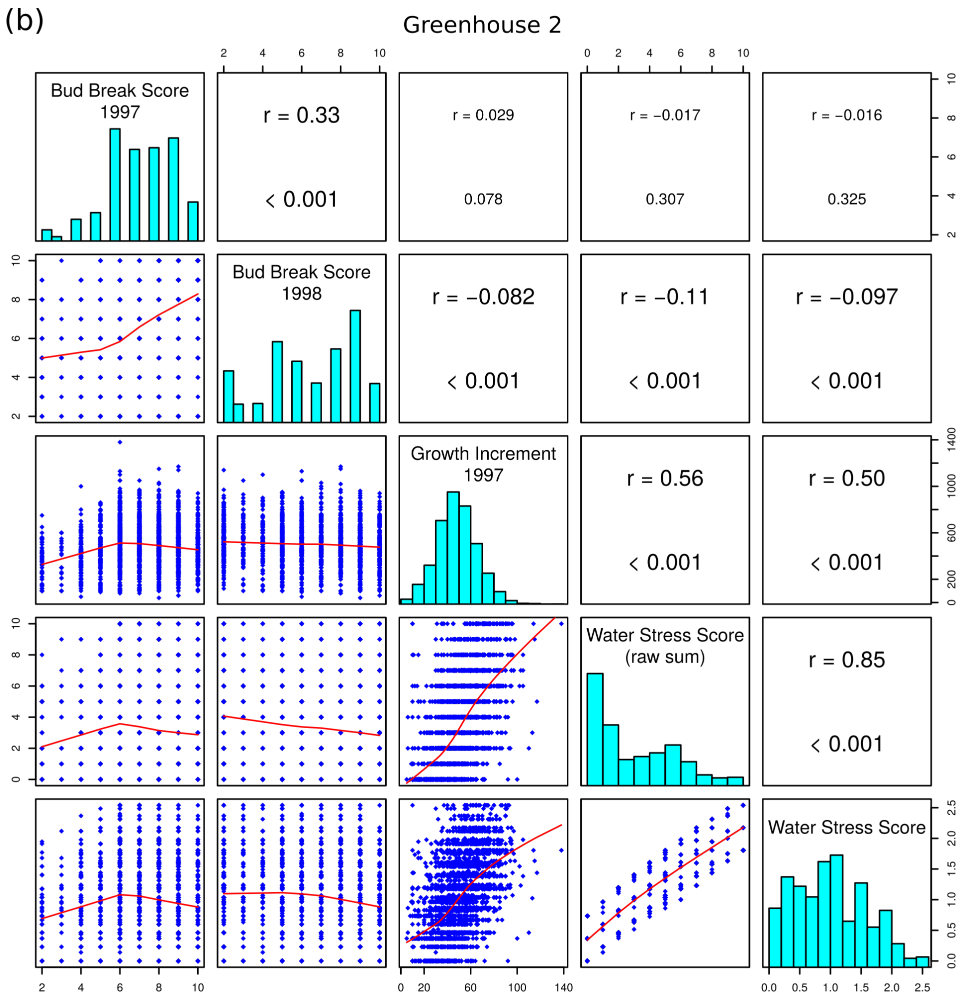

Figure S2: Raw seedling trait values and the correlation between them recorded in Greenhouse 1 **(a)** and Greenhouse 2 **(b)**. Seedlings originated from 16 silver fir (*Abies alba*) populations across the French Mediterranean Alps. Panels in the upper triangles show the Pearson correlation and the p-value from the correlation test. Significant correlations (p-value < 0.05) are shown in larger fonts. Panels in the lower triangle show a scatter plot between the two variables with a smooth curve in red fitted using the *lowess* function in R. Panels in the diagonal show the distribution the variables as histograms.

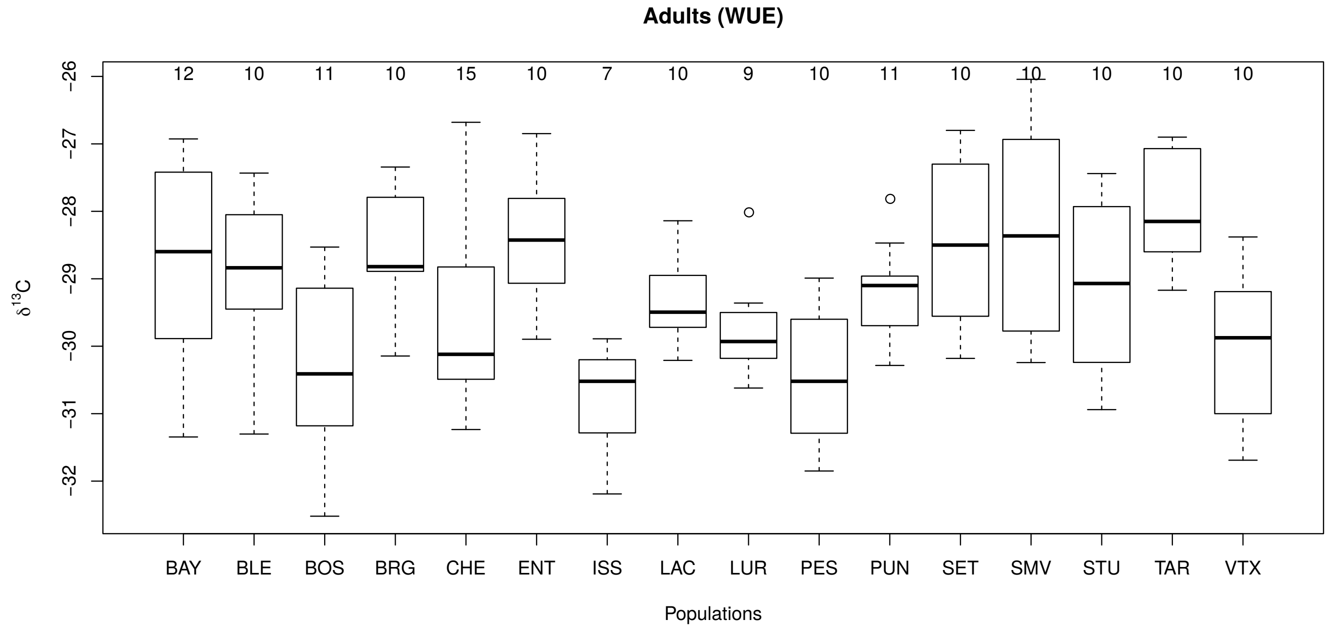

Figure S3: δ^{13}C across the 16 populations. The number of observations is indicated on the top of the figure above each box. δ^{13}C was measured from 2016 grown adult tree needle material.

Appendix A: Use of the greenhouse phenotype data in Sagnard et al. (2002), Roschanski et al. (2016) and in this study

In this study we analyzed observations from a greenhouse experiment run at the experimental forest nursery located in Milles (Southern France, Fig. 1) between 1995 and 1999. Part of this data have been analyzed and published in previous studies (Sagnard et al., 2002; Roschanski et al., 2016). The aim of this note is to highlight the differences between the previously published studies and this study.

Questions

Sagnard et al. (2002) aimed at contrasting patterns of variation across 16 silver fir populations using isozymes, adaptive traits and monoterpenes. In particular, the authors were interested if populations cluster according to previously identified phytosociological and phytoecological groups (Barbero and Bono, 1970), namely Sub-Mediterranean Alpine group, Intermediate Alpine group, and Ligurian Alpine group.

Roschanski et al. (2016) advocated an integrative landscape genetics approach to study the adaptive response of silver fir at four pairs of populations situated at low and high altitudes. Their aim was to detect candidate gene SNP loci that are unusually differentiated between populations, and/or whose allele frequencies are associated with abiotic or biotic factors. Further, they took advantage of the greenhouse data from Sagnard et al. (2002), generated by the same research group, and tested for adaptive trait divergence to provide complementary evidence for the genome scan methods.

Data

Seventeen provenances were tested in the greenhouse experiment, from which Sagnard et al. (2002) used 16, Roschanski et al. (2016) four, and the present study 16 (Table A1). Eighteen observations were recorded during the five years of the experiment from which Sagnard et al. (2002), Roschanski et al. (2016) and the present study used and derived different traits (see Table A2 for details).

Analyses and conclusions

Sagnard et al. (2002) demonstrated that the effect of family and population were significant using an two-way-ANOVA and estimated that the variance explained by the factor "population" was between 6.838 (Water Stress Score, sum) and 17.926 (Height 1999) (Table 3 in Sagnard et al. (2002)). The highest population differentiation for height is in agreement with our findings. Then, Sagnard et al. (2002) used Ward's method of hierarchical clustering to group populations according to trait values (all traits confounded), and found that population groups did not agree with geographical or ecological groups (Figure 2 in Sagnard et al. (2002)). In contrast, isozyme and, even more, monoterpene markers provided population clusters that agreed well with previously identified phytosociological and phytoecological groups (Figure 3 and Table 5 in Sagnard et al. (2002)). Since the authors found neither ecological nor geographical differentiation patterns for adaptive traits, they evoked five potential explanations among which two were privileged. First, considerable environmental variation at the local scale produce greater differentiation within than among populations. Second, the ecological

Table A1: 17 silver fir (*Abies alba* Mill.) populations used in the experimental forest nursery located in Milles (Southern France, Fig. 1) and their use in subsequent studies.

Population	Present study	Sagnard et al. (2002)	Roschanski et al. (2016)
BAY	yes	yes	-
BEU	yes	yes	-
BLE	yes	yes	-
BOS	yes	yes	-
BRG	yes	yes (BRI)	-
CHE	yes	yes	-
ENT	yes	yes	-
ISS	yes	yes (LAB)	yes
LAC	yes	yes	-
LUR	yes	yes	yes
PES	yes	yes	-
PUN	yes	-	-
SET	yes	yes	-
SMV	yes	yes	-
STU	yes	yes	-
TAR	yes	yes	-
TUR	-	yes	yes (VES)
VTX	yes	yes	yes

descriptors taken into account do not represent selective forces that the studied species has to face. In the light of the present study, we can confirm their conclusions.

Roschanski et al. (2016) first tested for differences between populations and performed a Q_{ST}-F_{ST} test for each trait. They found an overall weak evidence for adaptive divergence at the studied traits, which was likely due to the small number of populations. Further, they tested the response to water stress at each date independently, which gives less stable variables than the weighted some of scores used in this study. Nevertheless, their findings with four populations contain some remarkable similarities with ours. The Q_{ST}-F_{ST} test was significant for bud break only at the first observation (in 1997) and for Growth Increment (see Table 2 in Roschanski et al. (2016), and note that there is a typo in Roschanski et al. (2016), they analyzed Growth increment in 1997 and not in 1999; there was no growth observation in 1999). Further, they noticed that LUR had the slowest growth and highest resistance to water stress, however, they could not generalize their findings with four populations only.

Table A2: Traits recorded in the greenhouse experiment performed at the experimental forest nursery located in Milles (see Fig. 1) and their use in subsequent studies. See Table 2 for trait definitions.

Year	Period	Greenhouse	Trait	Present study	Sagnard et al. (2002)	Roschanski et al. (2016)
1995	-	1 and 2	Weight of 1000 seeds	Raw	Raw	-
1997	1 April	1 and 2	Bud Break Score	Sum of scores	-	Raw
	15 April	1 and 2	Bud Break Score	Sum of scores	Raw	Raw
	Autumn	1 and 2	Growth Increment	Raw	-	Raw
	Autumn	1	Height	Raw	1998 height increment	-
1998	3 April	1 and 2	Bud Break Score	Sum of scores	Raw	Raw
	17 April	1 and 2	Bud Break Score	Sum of scores	-	Raw
	5 June	2	Water Stress Score	Weighted sum of scores	Sum of scores	Raw
	9 June	2	Water Stress Score	Weighted sum of scores	Sum of scores	Raw
	12 June	2	Water Stress Score	Weighted sum of scores	Sum of scores	-
	15 June	2	Water Stress Score	Weighted sum of scores	Raw, Sum of scores	-
	17 June	2	Water Stress Score	Weighted sum of scores	Sum of scores	Raw
	22 June	2	Water Stress Score	Weighted sum of scores	Sum of scores	Raw
	Autumn	1	Height	Raw	1999 height increment	-
1999	March	1	Frost damage	-	Raw	-
	1 April	1	Bud Break Score	Sum of scores	-	Raw
	13 April	1	Bud Break Score	Sum of scores	-	Raw
	Autumn	1	Height	Raw	Raw	-

Appendix B: Inference of population structure using the Bayesian clustering algorithm implemented in the software *Structure*

Population genetic structure of the 16 silver fir (*Abies alba*) populations across the Mediterranean Alps was inferred using 357 SNP loci. We used the software *Structure Structure* v.2.3.4 (Pritchard et al., 2000) and the admixture model with correlated allele frequencies of the (Falush et al., 2003). We also included sampling location information to improve clustering performance ("locprior model", Hubisz et al. (2009)). We estimated the prior population allele frequency parameter (λ) from the data to account for the fact that SNPs often have rare minor alleles. We estimated λ using K=1 to avoid non-identifiability with the other hyper-parameters (λ, α, F). λ was consistently around 0.65 across ten repeated runs (range: 0.63-0.66, median: 0.65). Then, we tested K values from 1 to 19 using ten independent Markov chains for each K, and 500,000 burn-in iterations and 500,000 iterations for estimation of the membership coefficients. Different numbers of clusters (K) were compared with *StructureHarvester* (Earl and vonHoldt, 2012) using the $LnPr(X|K)$ and Evanno et al.'s (2005) method. Admixture coefficients were averaged across ten repeated runs using CLUMPP v.1.1.2 Jakobsson and Rosenberg (2007) using an exhaustive search for K$\leq$3, the Greedy algorithm for any K>3, and large-K-Greedy for K$\geq$5.

We obtained the highest support for the presence of six (log-likelihood method) and four (Evanno method) genetic clusters across the 16 silver fir populations (Fig. A1a). The population from the island of Corsica (PUN) was the most different from the other populations; in fact, a separation between Corsican and mainland populations was already clear with K=2 (Fig. A1b). With increasing K values, the genetic structure of the mainland populations was characterized by isolation-by-distance both from east to west and from south to north (Fig. A1b, Fig. 1a).

The mainland population TAR show a peculiar separation from the other population, with K=5 and 6 Fig. A1b), which is also confirmed by *RAFM* (Fig. 1b). Both analyses suggests that TAR is a cluster on its own, but with substantial admixture from neighboring clusters. TAR is situated in a warm and dry southern slope, which is a very surprising location for silver fir. We suggest that TAR could have eventually been a micro-refugium, where the species survived during the last glacial maximum. However, further studies would be needed to confirm this hypotheses.

(a)

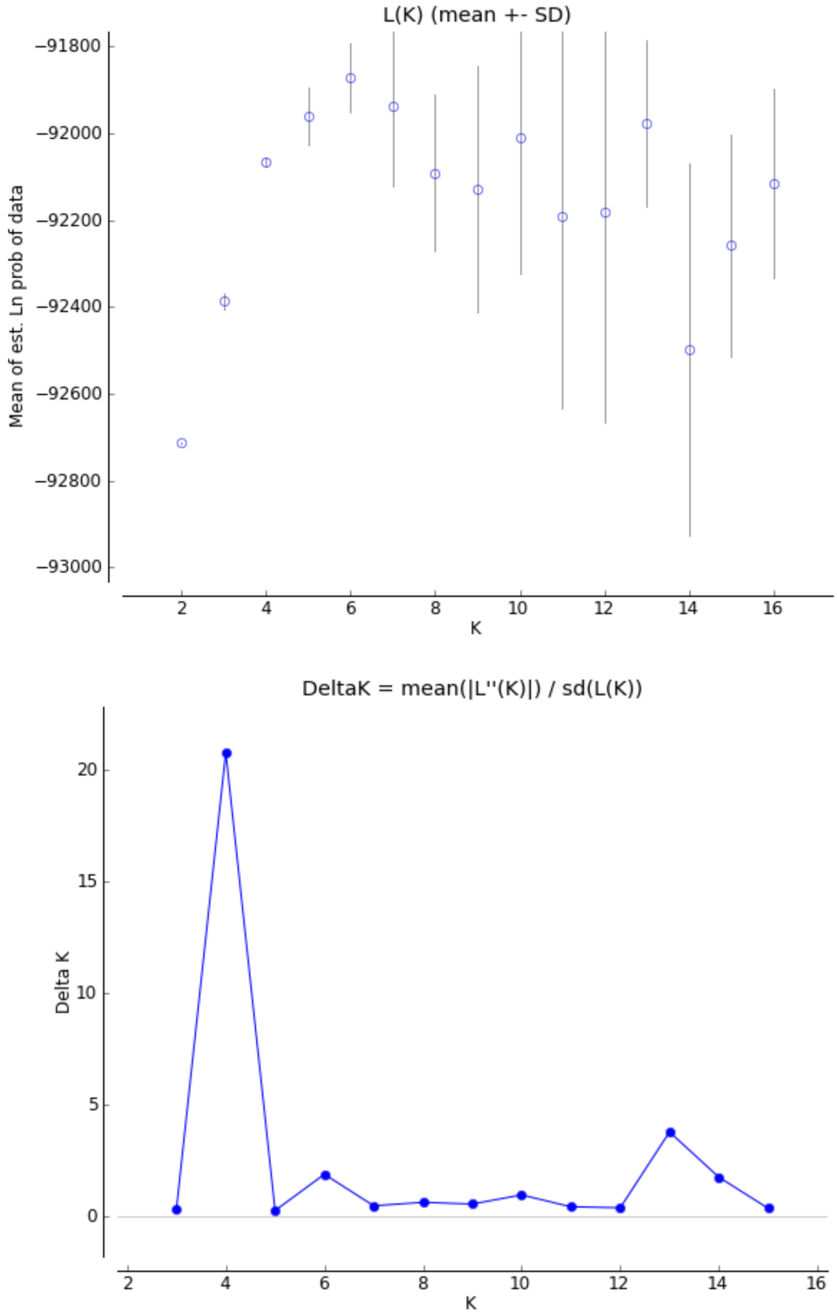
L(K) (mean +- SD)
Mean of est. Ln prob of data
−91800
−92000
−92200
−92400
−92600
−92800
−93000
2
4
6
8
10
12
14
16
K
DeltaK = mean(|L''(K)|) / sd(L(K))
Delta K
20
15
10
5
0
2
4
6
8
10
12
14
16
K

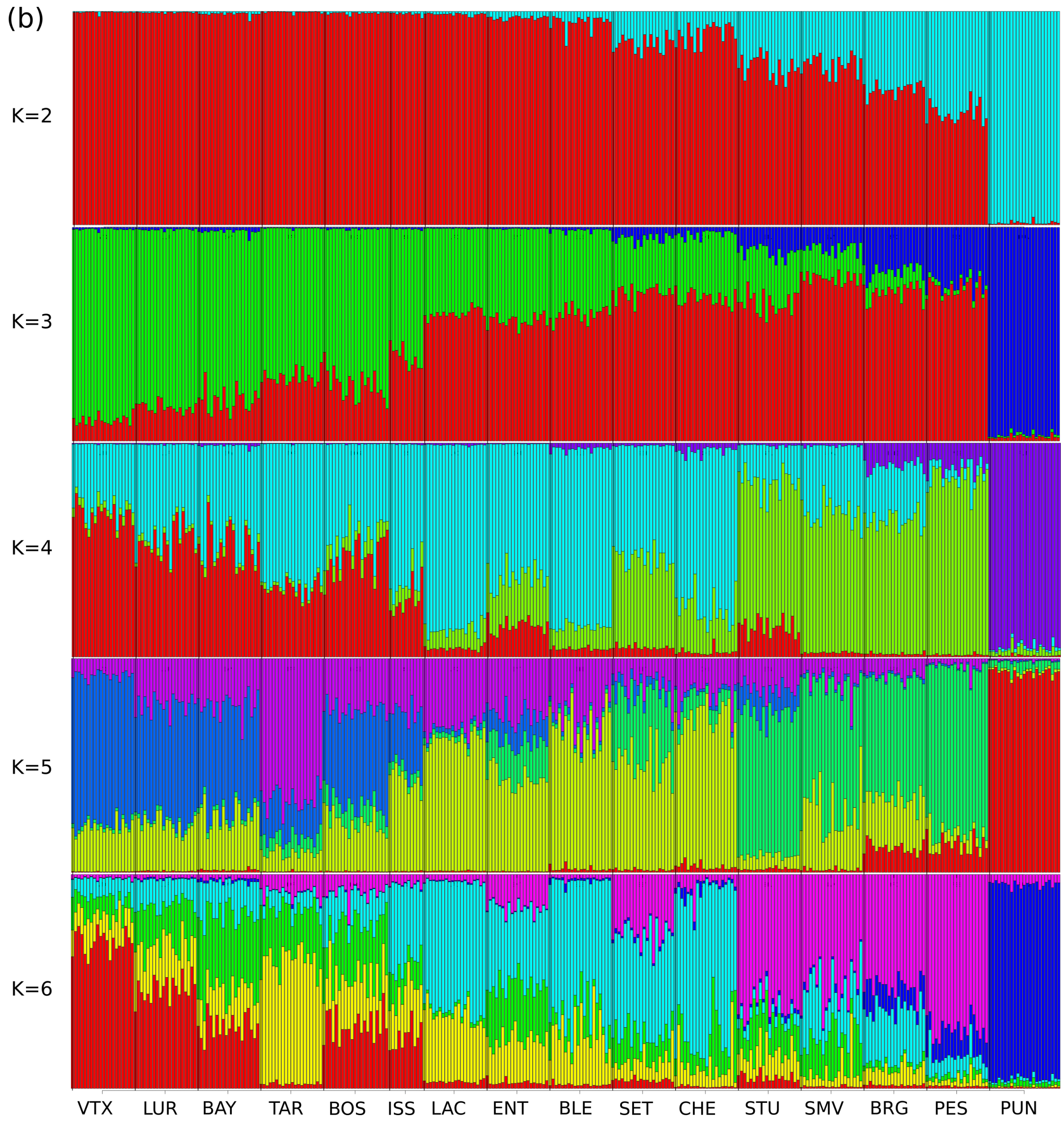

Figure A1: (a) Log likelihood of the data and the delta K values (Evanno et al., 2005) for K values between two and 16. **(b)** Proportion of ancestry of each sampled tree (colored vertical bars) from each of the assumed genetic clusters (K). Each cluster is indicated with a different color and colors are arbitrary between different values of K. Results for K values between one and six are shown.

Appendix C: Estimating variance components using the 'animal model'

We used the animal model (Henderson, 1975; Kruuk, 2004) implemented in R-ASReml 3 (Butler et al., 2009) to partition the trait variance into different components. From these, we estimated the heritability and evolutionary potential of individual traits, and the genetic correlations between traits. The animal model is a type of mixed-effects model defined at the level of each individual seedling, which estimate of the additive genetic variance (V_A) of a trait or the additive genetic covariance between two traits (COV_A) from the covariance between relatives. We ran a separate model for each of the 11 traits (Table 2) that included block as a fixed effect (with two or four values depending if one or both greenhouses were considered), and seed weight, population, and seedling as random effects, and a random residual error for each seedling. We constructed the pedigree assuming seedlings from the same mother tree are half-sibs (see main text for the equation). We constructed the inverse kinship matrix using the function *asreml.Ainverse*.

0.1 Univariate models

Model checks

We performed several model checks and tests of robustness to assumptions. First, we tested the significance of each component. We tested if environmental heterogeneity generated by the experimental designed contributed to the trait variance by comparing models with and without block effect using a Wald-test (*wald.asreml*). We also tested for the significance of the random effects using a likelihood ratio test between models with and without seed weight and population, and also tested significance of including the pedigree itself. Second, we repeated the analysis for demographically homogeneous regions to test if V_A is homogeneous across the study region. Third, we tested the effect of assuming a purely outcrossing mating system.

Model selection

Block, i.e./ environmental heterogeneity within the greenhouses, had a significant effect on the variation of most traits, but on Bud Break Score 1998 and 1999, and Height 1998 (Table A3). For three traits, Bud Break Score 1997 and 1998, and Growth Increment 1997, we were also able to compare the effect of the greenhouse. We found that variance components were similar when taking all data or just one of the greenhouses, with slightly higher estimates for one greenhouse only, and especially for Greenhouse 2 (Table A3, Fig. 3). The pedigree ("Family") always explained a large percentage of the trait variation and a model with pedigree was always better than a model without it (Table A3). We tested for the effect of seed weight to assess if genetic or non-genetic maternal effects explain trait variation. Seed weight explained a negligible (at the maximum 0.027%, Fig. A2), yet significant part of trait variation in growth traits, i.e. Growth Increment and Height, but not in phenology (Table A3). Population of origin also explained a significant part of the trait variation (Table A3). The proportion of variance explained by population varied between 9.8% (Height 1998) and 0.7% (Bud Break Score 1998) with a mean of 5% across all traits (Fig. A2).

Is V_A homogeneous across the study region?

Estimating the evolutionary potential using a model that incorporates all observations implicitly assumes that the 16 populations have a common V_A. This assumption may not hold

Table A3: Model comparison of 11 silver fir (*Abies alba*) seedling traits measured in the greenhouse using an animal model implemented in *ASreml-R*. Seedlings were grown in two greenhouses each comprising two blocks. Populations and families were randomized across greenhouses and blocks. N indicates the number the observations. The role of Block (fixed effect) was tested using a Wald-test, while the role of Family (i.e. the pedigree), and Seed weight and Population (random effects) were tested using a likelihood ratio test by excluding each of these variables one-by-one.

Trait	Greenhouse	N	Block	Family	Seed weight	Population
Bud Break Score 1997	1	3862	0.0020	< 0.0001	> 0.9999	< 0.0001
	2	3883	< 0.0001	< 0.0001	> 0.9999	< 0.0001
Bud Break Score 1998	1	1566	0.2029	< 0.0001	> 0.9999	0.1356
	2	2935	0.0017	< 0.0001	0.3801	< 0.0001
Bud Break Score 1999	1	3592	0.3522	< 0.0001	0.0784	0.0002
Growth Increment 1997	1	3208	< 0.0001	< 0.0001	< 0.0001	< 0.0001
	2	3620	< 0.0001	< 0.0001	< 0.0001	< 0.0001
Height 1997	1	898	0.0006	0.0071	0.3228	0.0002
Height 1998	1	1683	0.2588	0.0001	0.0482	< 0.0001
Height 1999	1	3705	< 0.0001	< 0.0001	0.0004	< 0.0001
Water Stress Score 1998	2	3620	< 0.0001	< 0.0001	< 0.0001	< 0.0001

because populations were selected from an environmentally and demographically heterogeneous region. We tested the hypothesis of homogeneity in V_A across the study region by running the univariate models for the three main genetic clusters identified (see main text). The three clusters were: BOS, BAY, LUR, ISS, VTX (North-Western cluster, 2496 observations); SET, ENT, LAC, BLE, CHE (Central cluster, 2639 observations); and PES, BRG, STU and SMV (Eastern clusters, 1883 observations). TAR and PUN were excluded from this regional analysis because they did not clearly belong to any of the three clusters (Fig. 1). The regional analysis revealed the same relative differences between the traits in h^2 and CV_A, however, estimates were often higher (i.e. inflated) most likely owing to a smaller sample size (Fig. A3).

What is the effect of the mating system on V_A?

Silver fir is a predominantly outcrossing species, thus we assumed throughout this paper that all seedlings from the same mother tree are half-sibs. However, the mating system in silver fir is likely composed of a mixture of outcrossing, bi-parental inbreeding and selfing. We do not have information about the outcrossing rate at the 16 sites, so we could not objectively correct for this potential bias. Nevertheless, we could estimate the h^2 and CV_A by assuming that all seedlings from the same mother tree were issued from selfing. With this analysis, we could set a biological lower bound on the quantitative genetic parameters. Fig. A4 shows that assuming 100% selfing mother trees the relative differences in evolutionary potential stay similar among traits, however, the absolute values would be three to four times lower.

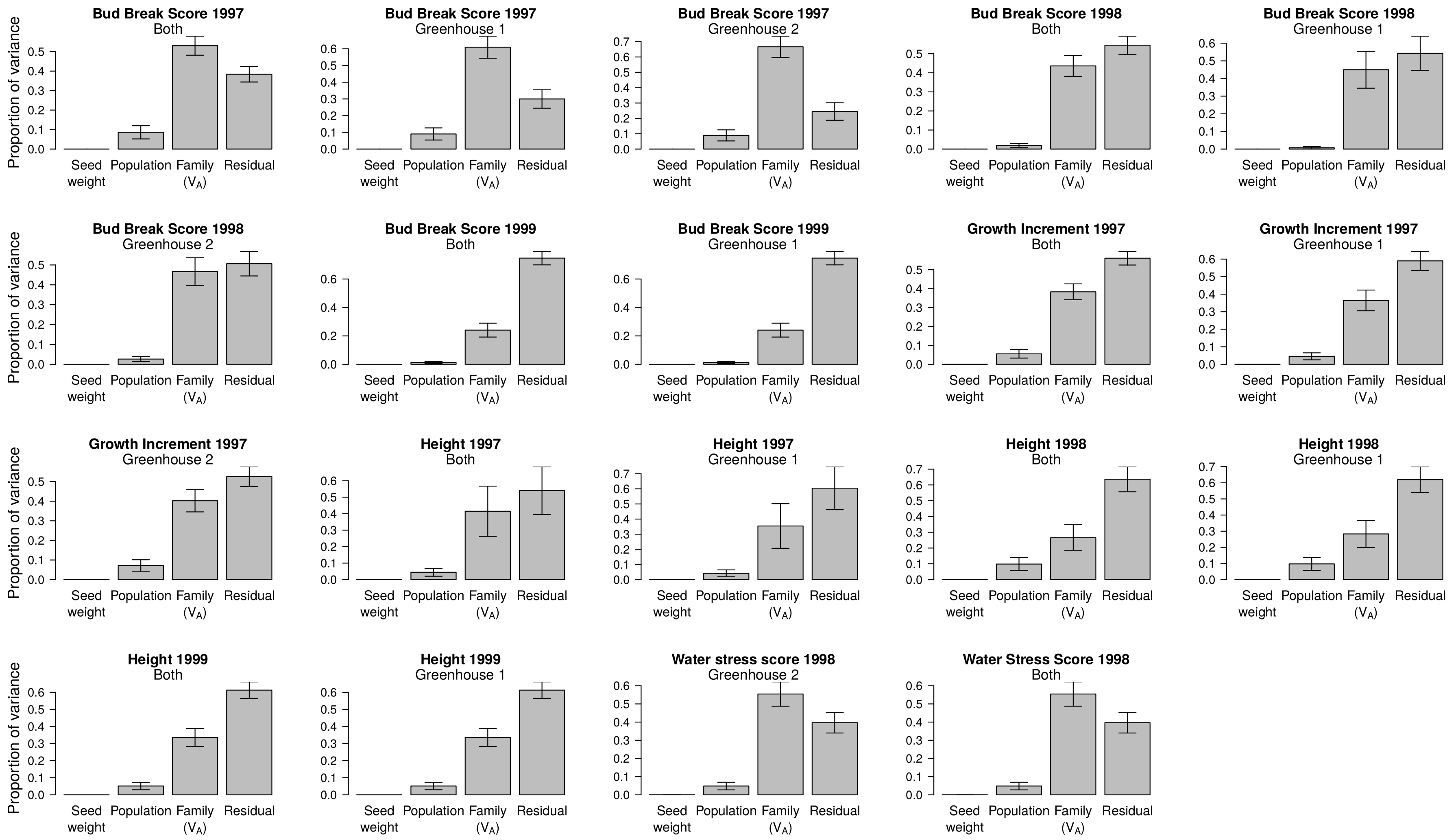

Figure A2: Variance components estimated using a mixed-effects model called the animal model implemented in *ASreml-R* including block as a fixed effect, and seed weight and population, and family structure (i.e. the pedigree) as random effects. V_A stands for the additive genetic variance, which is the trait variance due to resemblance between family members.

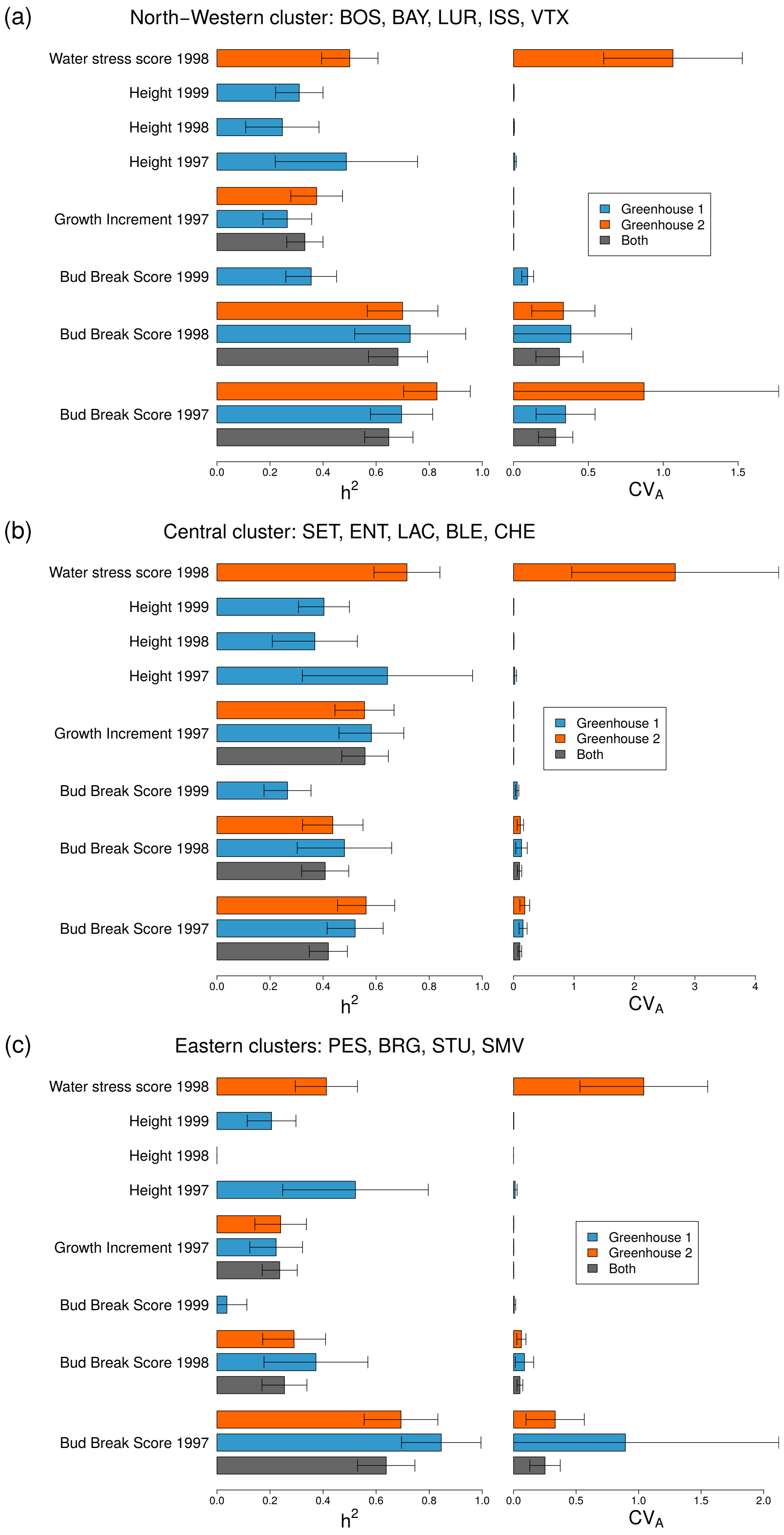

Figure A3: (see legend on the next page)

Figure A3 Heritability (h^2) and additive genetic coefficient of variation (CV_A) of 11 traits measured on silver fir (*Abies alba*) seedling in the greenhouse. The full data set comprising two greenhouses and 8199 observations were divided into three groups according to the genetic and geographic clustering of the populations. **(a)** North-Western cluster (N=2496), **(b)** Central cluster (N=2639), and **(c)** Eastern cluster (N=1883). Two genetically isolated populations, TAR and PUN, were excluded from this analysis. Not all traits were scored in both greenhouses. Greenhouse 2 received a water stress treatment in 1998 after which only Water Stress Score was recorded.

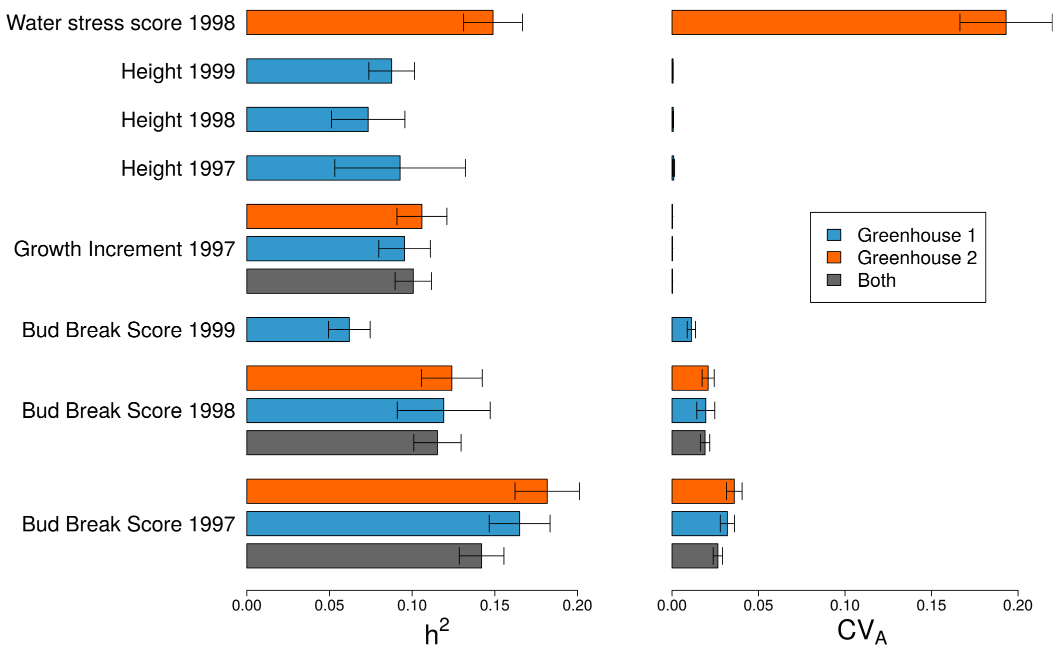

Figure A4: The lower bound on heritability (h^2) and additive genetic coefficient of variation (CV_A) estimated assuming that all seedlings were issued from selfing. 11 traits measured on silver fir (*Abies alba*) seedling in the greenhouse. The experiment consisted of two greenhouses and counted 8199 observations. Not all traits were scored in both greenhouses. Greenhouse 2 received a water stress treatment in 1998 after which only Water Stress Score was recorded.

Multivariate models

The experiment consisted of two greenhouses, and not all traits were scored in both greenhouses (Table 1). Thus, we were not able to run multivariate models to estimate the additive genetic covariance between all pairs of traits. We were able to run three separate multivariate models that best exploited the available data. First, a two-trait model with Bud Break Score 1997 and Growth Increment 1997 including observations from both greenhouses (N=6815). Second, a four-trait model with Bud Break Score 1997 and 1999, Growth Increment 1997, and Height 1999 including observations from Greenhouse 1 (N=3481). Third, a three-trait model with Bud Break Score 1997 and Growth Increment 1997, and Water Stress Score 1998 including observations from Greenhouse 2 (N=2888). Note that the sample sizes are less than in Table 1 because data sets for these models had to contain non-missing observations for multiple traits at a time. Using these multivariate models we estimated the genetic correlations between traits as the additive genetic covariance between traits (COV_A) standardized by the product of the additive genetic standard deviations ($\sqrt{V_A}$) for the two trait.

Genetic correlations

Multivariate animal models revealed several significant genetic correlations between traits. For one trait pair, Bud Break Score 1997 and Growth Increment 1997, we obtained estimates of the genetic correlations with all three models. The three estimates were similar and non-significant, so we show only the estimate obtained with the largest sample size (i.e. with both greenhouses). The strongest, positive and highly significant, genetic correlation was observed between Growth Increment 1997 and Water Stress Score 1998 (Fig. A5, see main text for discussions). Positive genetic correlations were also present between traits were measured in repeated years, such as between Bud Break Score in 1997 and 1999, or between Growth Increment 1997 and Height 1999 (Fig. A5). A negative genetic correlation was detected between Bud Break and growth traits: in 1999 seedlings that broke buds later stayed smaller by the end of the growing season, but also seedlings that had a lower Growth Increment in 1997 broke buds later in 1999 (Fig. A5).

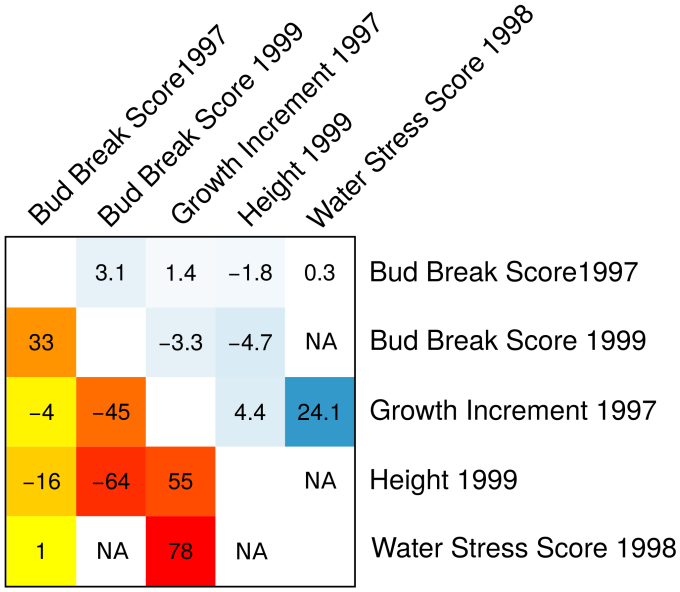

Figure A5: Genetic correlations estimated using the animal model implemented in *ASreml-R* including block as a fixed effect, and seed weight and population, and seedling (i.e. the pedigree) as random effects. The lower triangle shows the genetic correlations expressed as percentages, while the upper triangle the z-ratio (COV_A/SE). Absolute z-ratio higher than 2 indicates genetic correlations significantly different from zero. Correlations were estimated using Greenhouse 1 or 2 or both depending on where the traits were measured.

References

Barbero, M. and Bono, G. (1970). Les sapiniéres des Alpes Maritimes, da l'Authion à la Ligurie et de la Stura au Tanaro. *Veröff. Geobot. Inst. ETH Stiftung Rübel Zürich*, 43, 140–168.

Butler, D., Cullis, B. R., Gilmour, A., and Gogel, B. (2009). Asreml-r reference manual. *The State of Queensland, Department of Primary Industries and Fisheries, Brisbane.*

Csilléry, K., Ovaskainen, O., Sperisen, C., Buchmann, N., Widmer, A., and Gugerli, F. (2020). Adaptation to local climate in multi-trait space: evidence from silver fir (*Abies alba* Mill.) populations across a heterogeneous environment. *Heredity*, 124, 77–92.

Earl, D. A. and vonHoldt, B. M. (2012). STRUCTURE HARVESTER: a website and program for visualizing STRUCTURE output and implementing the Evanno method. *Conservation Genetics Resources*, 4, 359–361.

Evanno, G., Regnaut, S., and Goudet, J. (2005). Detecting the number of clusters of individuals using the software STRUCTURE: a simulation study. *Molecular Ecology*, 14, 2611–2620.

Falush, D., Stephens, M., and Pritchard, J. K. (2003). Inference of population structure using multilocus genotype data: linked loci and correlated allele frequencies. *Genetics*, 164, 1567–1587.

Harris, I., Jones, P., Osborn, T., and Lister, D. (2014). Updated high-resolution grids of monthly climatic observations–the CRU TS3.10 Dataset. *International Journal of Climatology*, 34, 623–642.

Henderson, C. R. (1975). Best linear unbiased estimation and prediction under a selection model. *Biometrics*, pages 423–447.

Hengl, T., de Jesus, J. M., Heuvelink, G. B., Gonzalez, M. R., Kilibarda, M., Blagotić, A., Shangguan, W., Wright, M. N., Geng, X., Bauer-Marschallinger, B., et al. (2017). SoilGrids250m: Global gridded soil information based on machine learning. *PLoS ONE*, 12, e0169748.

Hubisz, M. J., Falush, D., Stephens, M., and Pritchard, J. K. (2009). Inferring weak population structure with the assistance of sample group information. *Molecular Ecology Resources*, 9, 1322–1332.

Jakobsson, M. and Rosenberg, N. A. (2007). CLUMPP: a cluster matching and permutation program for dealing with label switching and multimodality in analysis of population structure. *Bioinformatics*, 23, 1801–1806.

Karger, D. N., Conrad, O., Böhner, J., Kawohl, T., Kreft, H., Soria-Auza, R. W., Zimmermann, N. E., Linder, H. P., and Kessler, M. (2017). Climatologies at high resolution for the Earth's land surface areas. *Scientific Data*, 4, 170122.

Karhunen, M., Ovaskainen, O., Herczeg, G., and Merilä, J. (2014). Bringing habitat information into statistical tests of local adaptation in quantitative traits: A case study of nine-spined sticklebacks. *Evolution*, 68, 559–568.

Kruuk, L. E. B. (2004). Estimating genetic parameters in natural populations using the 'animal model'. *Proc. R. Soc. Lond. B*, 359, 873–890.

Ovaskainen, O., Karhunen, M., Zheng, C., Arias, J. M. C., and Merilä, J. (2011). A new method to uncover signatures of divergent and stabilizing selection in quantitative traits. *Genetics*, 189, 621–632.

Pritchard, J. K., Stephens, M., and Donnelly, P. J. (2000). Inference of population structure using multilocus genotype data. *Genetics*, 155, 945–959.

Roschanski, A. M., Csilléry, K., Liepelt, S., Oddou-Muratorio, S., Ziegenhagen, B., Huard, F., Ullrich, K. K., Postolache, D., Vendramin, G. G., and Fady, B. (2016). Evidence of divergent selection for drought and cold tolerance at landscape and local scales in *Abies alba* Mill. in the French Mediterranean Alps. *Molecular Ecology*, 25, 776–794.

Sagnard, F., Barberot, C., and Fady, B. (2002). Structure of genetic diversity in *Abies alba* Mill. from southwestern Alps: Multivariate analysis of adaptive and non-adaptive traits for conservation in France. *Forest Ecology and Management*, 157, 175–189.
